# Functional Annotation of *ESR1* Gene Fusions in Estrogen Receptor-Positive Breast Cancer

**DOI:** 10.1016/j.celrep.2018.07.009

**Published:** 2018-08-07

**Authors:** Jonathan T. Lei, Jieya Shao, Jin Zhang, Michael Iglesia, Doug W. Chan, Jin Cao, Meenakshi Anurag, Purba Singh, Xiaping He, Yoshimasa Kosaka, Ryoichi Matsunuma, Robert Crowder, Jeremy Hoog, Chanpheng Phommaly, Rodrigo Goncalves, Susana Ramalho, Raquel Mary Rodrigues Peres, Nindo Punturi, Cheryl Schmidt, Alex Bartram, Eric Jou, Vaishnavi Devarakonda, Kimberly R. Holloway, W. Victoria Lai, Oliver Hampton, Anna Rogers, Ethan Tobias, Poojan A. Parikh, Sherri R. Davies, Shunqiang Li, Cynthia X. Ma, Vera J. Suman, Kelly K. Hunt, Mark A. Watson, Katherine A. Hoadley, E. Aubrey Thompson, Xi Chen, Shyam M. Kavuri, Chad J. Creighton, Christopher A. Maher, Charles M. Perou, Svasti Haricharan, Matthew J. Ellis

**Affiliations:** 1 Department of Medicine, Lester and Sue Smith Breast Center, Baylor College of Medicine, Houston, TX 77030, USA; 2 Interdepartmental Graduate Program in Translational Biology and Molecular Medicine, Baylor College of Medicine, Houston, TX 77030, USA; 3 Department of Medicine, Washington University in St. Louis, St. Louis, MO 63110, USA; 4 Siteman Cancer Center, Washington University in St. Louis, St. Louis, MO 63110, USA; 5 Cancer Biology Division, Department of Radiation Oncology, Washington University in St. Louis, St. Louis, MO 63110, USA; 6 Institute for Informatics (I^2^), Washington University in St. Louis, St. Louis, MO 63110, USA; 7 Department of Molecular and Cellular Biology, Baylor College of Medicine, Houston, TX 77030, USA; 8 Department of Genetics, Lineberger Comprehensive Cancer Center, University of North Carolina, Chapel Hill, NC 27599, USA; 9 Department of Breast and Endocrine Surgery, Kitasato University School of Medicine, Sagamihara, Kanagawa 252-0375, Japan; 10 First Department of Surgery, Hamamatsu University School of Medicine, Hamamatsu, Shizuoka 431-3192, Japan; 11 Department of Obstetrics and Gynecology, University of São Paulo School of Medicine (FMUSP), Cerqueira César, São Paulo 01246-903, Brazil; 12 Department of Obstetrics and Gynecology, Faculty of Medical Science, State University of Campinas - UNICAMP, Campinas, São Paulo 13083-970, Brazil; 13 Queens’ College, University of Cambridge, Cambridge CB3 9ET, UK; 14 Division of Solid Tumor Oncology, Department of Medicine, Memorial Sloan Kettering Cancer Center, New York, NY 10065, USA; 15 Human Genome Sequencing Center, Department of Molecular and Human Genetics, Baylor College of Medicine, Houston, TX 77030, USA; 16 University of Texas Southwestern Medical Center, Dallas, TX 75390, USA; 17 School of Medicine, Baylor College of Medicine, Houston, TX 77030, USA; 18 Alliance Statistical Center, Mayo Clinic, Rochester, MN 55905, USA; 19 Department of Breast Surgical Oncology, MD Anderson Cancer Center, Houston, TX 77030, USA; 20 Department of Pathology and Immunology, Washington University in St. Louis, St. Louis, MO 63110, USA; 21 Department of Cancer Biology, Mayo Clinic Comprehensive Cancer Center, Jacksonville, FL 32224, USA; 22 Department of Medicine, Baylor College of Medicine, Houston, TX 77030, USA; 23 The McDonnell Genome Institute, Washington University in St. Louis, St. Louis, MO 63108, USA; 24 These authors contributed equally; 25 Lead Contact

## Abstract

RNA sequencing (RNA-seq) detects estrogen receptor alpha gene (*ESR1*) fusion transcripts in estrogen receptor-positive (ER^+^) breast cancer, but their role in disease pathogenesis remains unclear. We examined multiple *ESR1* fusions and found that two, both identified in advanced endocrine treatment-resistant disease, encoded stable and functional fusion proteins. In both examples, ESR1-e6>YAP1 and ESR1-e6>PCDH11X, *ESR1* exons 1–6 were fused in frame to C-terminal sequences from the partner gene. Functional properties include estrogen-independent growth, constitutive expression of ER target genes, and anti-estrogen resistance. Both fusions activate a metastasis-associated transcriptional program, induce cellular motility, and promote the development of lung metastasis. ESR1-e6>YAP1- and ESR1-e6>PCDH11X-induced growth remained sensitive to a CDK4/6 inhibitor, and a patient-derived xenograft (PDX) naturally expressing the ESR1-e6>YAP1 fusion was also responsive. Transcriptionally active *ESR1* fusions therefore trigger both endocrine therapy resistance and metastatic progression, explaining the association with fatal disease progression, although CDK4/6 inhibitor treatment is predicted to be effective.

## In Brief

Lei et al. show that transcriptionally active estrogen receptor gene (*ESR1*) fusions identified from late-stage, treatment-refractory estrogen receptor-positive (ER+) breast cancer drive pan-endocrine therapy resistance and metastatic progression. Growth of breast tumors driven by *ESR1* fusions at primary and metastatic sties can be suppressed with a CDK4/6 inhibitor.

## Graphical Abstract

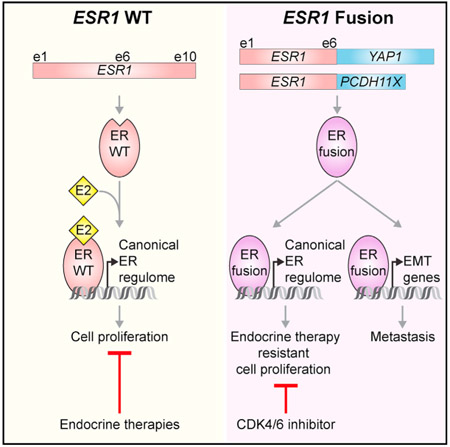

## INTRODUCTION

The etiology of endocrine therapy resistance in estrogen receptor-positive (ER^+^) breast cancer is complex (Ma et al., 2015) but includes acquired somatic mutations within the ligand-binding domain (LBD) of the estrogen receptor gene (*ESR1*) causing ligand-independent activation (Pejerrey et al., 2018). RNA sequencing (RNA-seq) has also identified multiple *ESR1* gene fusion events, but their role in endocrine therapy resistance and how they might be targeted therapeutically is unclear (Giltnane et al., 2017). The majority of *ESR1* fusion transcripts have been identified in primary breast cancer, and in some of these instances patients have high-grade disease and/or resistance to endocrine therapy (Giltnane et al., 2017; Veeraraghavan et al., 2014), implying some functionality. In some cases, up to five *ESR1* coding exons are included (exons 3–7), mostly fused out of frame but occasionally, and more interestingly, in frame. However, detailed characterization of the predicted chimeric proteins and a clear demonstration of a causal role for *ESR1* fusions in endocrine therapy resistance have been largely lacking.

Several years ago, our group described an unequivocal stable and functional *ESR1* fusion protein (Li et al., 2013). This was an in-frame fusion gene consisting of exons 1–6 of *ESR1* fused to C-terminal sequences from the Hippo pathway coactivator *YAP1* (ESR1-e6>YAP1), identified in a metastatic sample and matched patient-derived xenograft (PDX) from a patient with endocrine therapy-resistant disease. Limited functional characterization of ESR1-e6>YAP1 showed that the fusion protein drove resistance to endocrine therapy and estradiol-independent proliferation. Herein we build on our original report by contrasting the functional, transcriptional, and pharmacological properties of the ESR1-e6>YAP1 fusion with additional *ESR1* gene fusion events identified by RNA-seq of both early-stage and metastatic ER^+^ breast cancers.

## RESULTS

### Identification and Verification of In-Frame *ESR1* Gene Fusions

**Figure 1.**
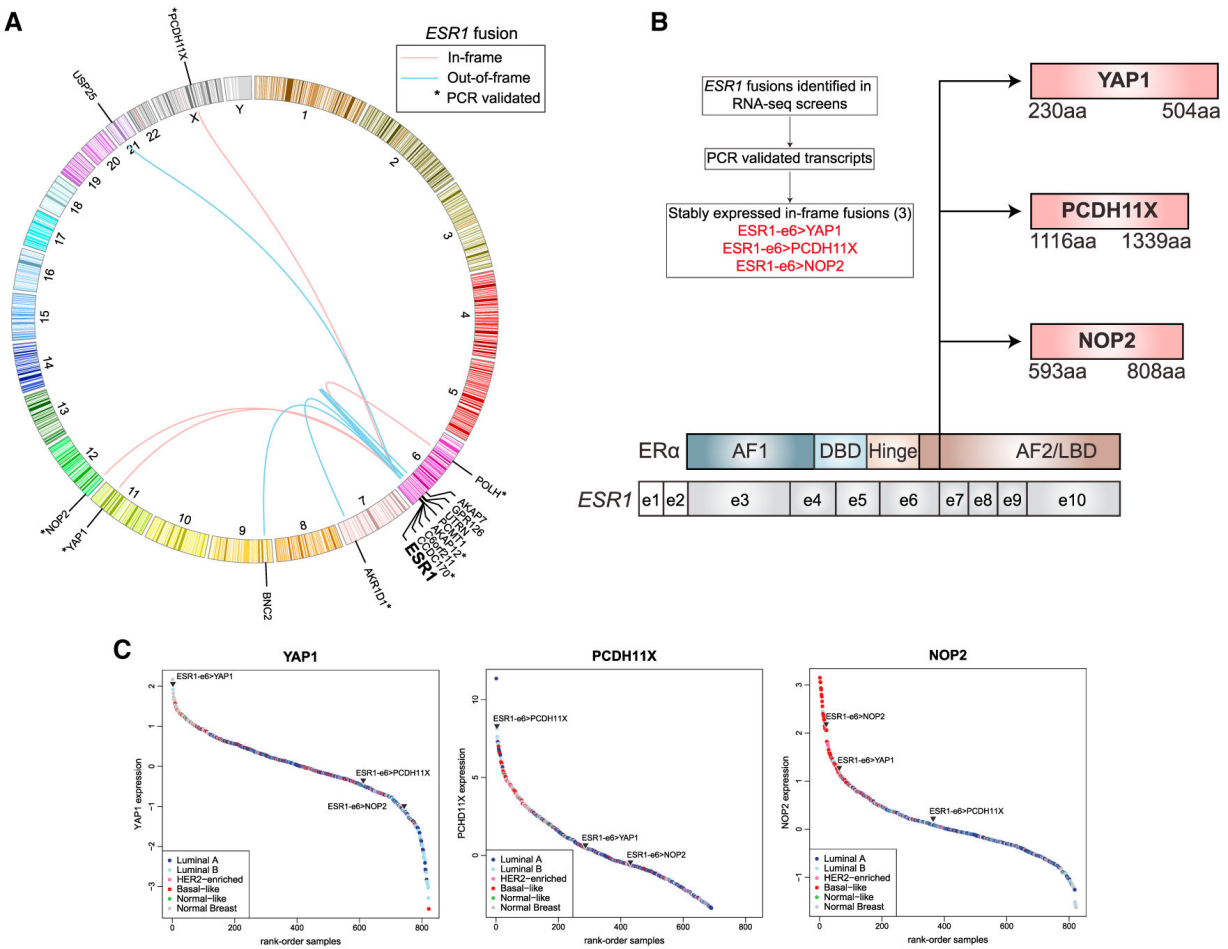
Identification and Verification of *ESR1* Fusions (A) Circos plot depicting *ESR1* fusion events from Table S1. In-frame *ESR1* fusions are depicted with a red line, and out-of-frame *ESR1* fusions depicted with a blue line. Asterisks denote PCR-validated transcripts (Figure S1). (B)Of eight *ESR1* fusions identified in (A) that were PCR validated, only three *ESR1* fusions produced stable, in-frame proteins (indicated in red): ESR1-e6>YAP1, ESR1-e6>PCDH11X, and ESR1-e6>NOP2. Illustration depicting in-frame *ESR1* fusions with *ESR1* codon structure shown at the bottom. Non-coding exons (e) 1 and 2 are shown as white boxes, and gray boxes depict exons encoding domains shown above. Vertical line indicates shared break points after exon 6 of *ESR1*. All depicted fusions retain exons encoding amino acids (aa) 1–365 of ER corresponding to the activation function 1 (AF1) domain, DNA-binding domain (DBD), the hinge region that includes the nuclear localization domain, and part of the activation function 2 (AF2)/ligand-binding domain (LBD). (C)RNA-seq determined rank-ordered expression of *YAP1, PCDH11X*, and *NOP2* from 728 TCGA breast tumor samples, shown as colored circles according to subtype. Triangles indicate ranked expression from indicated *ESR1* fusion containing sample among the TCGA breast samples. See also Figure S1.

A systematic screen was conducted to identify *ESR1* translocations in three datasets: 728 primary breast tumors from The Cancer Genome Atlas (TCGA) (Ciriello et al., 2015), 81 primary breast cancers from two neoadjuvant aromatase inhibitor (AI) clinical trials (Ellis et al., 2011; Olson et al., 2009), and 25 biopsy samples from patients with late-stage ER^+^ breast cancer (Figure 1A). From these analyses, 13 high-confidence *ESR1* fusion transcripts were identified in 10 ER^+^ samples from the TCGA dataset (Table S1). Five of these fusion events were between *ESR1* and *CCDC170* and were recently reported (Veeraraghavan et al., 2014). Of these, only 1 CCDC170 out-of-frame fusion included exon 5 (e5) of *ESR1* (ESR1-e5>CCDC170), thereby preserving sufficient *ESR1* sequence to bind DNA. A single TCGA case displayed evidence for three *ESR1* gene fusions: (1) a PCR-validated ESR1-e6 fused in frame to C-terminal sequences from *AKAP12* (ESR1-e6>AKAP12) (Figure S1); (2) a PCR-validated in-frame ESR1-e7 fusion involving the entire coding sequence of *POLH*, a DNA polymerase in the xeroderma pigmentosum gene family (ESR1-e7>POLH), and (3) an out-of-frame ESR1-e4>CCDC170 fusion.

From an RNA-seq screen of 81 primary, treatment-naive, ER^+^ breast cancers from two neoadjuvant AI clinical trials (Table S1, NeoAI Trials), two PCR-validated *ESR1* fusions were identified. The first was an in-frame fusion retaining the first six exons of *ESR1* (ESR1-e6) fused to C-terminal sequences of *NOP2*, a nucleolar protein (ESR1-e6>NOP2). The second fusion identified involved ESR1-e6 fused out of frame to *AKR1D1*, an aldo-keto reductase family member (ESR1-e6>AKR1D1). In the datasets of primary ER^+^ breast cancer examined, *ESR1* fusion events are relatively rare, occurring at ~2% frequency. The majority of these fusions are out of frame, and 42% of these fusion events (8 of 19) include sufficient *ESR1* exons to allow *ESR1* -specific nuclear binding.

To investigate *ESR1* fusion events in late-stage ER^+^ disease, RNA-seq data from 25 biopsy samples obtained from patients with advanced endocrine therapy refractory disease were examined (Table S1, Late Stage, and Table S2). These samples included the ESR1-e6>YAP1 sample we originally described, as it was drawn from this series (Li et al., 2013), and of these 25 samples, 2 harbored in-frame *ESR1* fusion events. The ESR1-e6>PCDH11X fusion was caused by ESR1-e6 fusion in frame with C-terminal sequences of protocadherin 11X. *PCDH11X* encodes for an atypical cell surface cadherin family member. The sample was a chest wall recurrence from a 49-year-old man who presented with locally advanced ER^+^ breast cancer and experienced progression on tamoxifen, letrozole/leuprolide, and fulvestrant before the sample was accrued.

**Figure 2.**
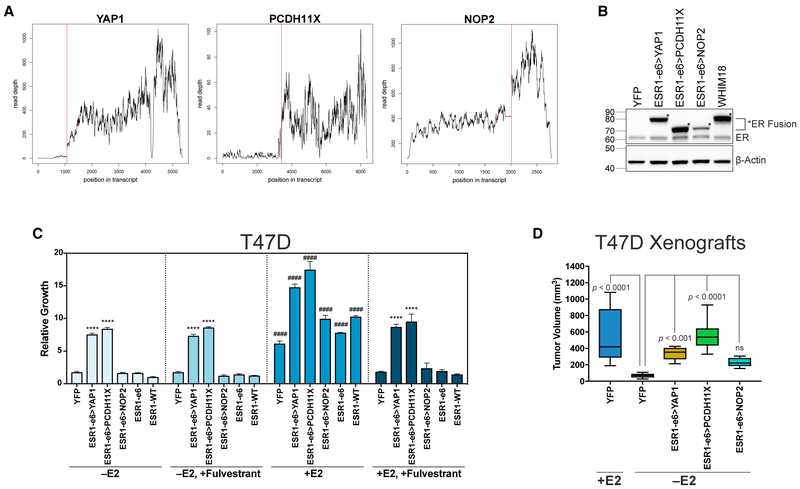
In-Frame *ESR1* Fusions from Endocrine-Refractory Disease Confer Estrogen-Independent and Fulvestrant-Resistant Growth of ER^+^ Breast Cancer Cells (A) RNA-seq mapped read depth was calculated across *YAP1, PCDH11X*, and *NOP2* genes in corresponding fusion containing tumors. Red line indicates fusion breakpoints. (B) Immunoblotting with an N-terminal ER antibody in hormone-deprived stable T47D and WHIM18 PDX. Asterisks indicate ER fusion. (C) Cell proliferation studies of hormone-deprived stable T47D cells (−E2), after fulvestrant treatment (−E2, +Fulvestrant), after E2 stimulation (+E2), or after E2 stimulation with fulvestrant treatment (+E2, +Fulvestrant). Bar graphs show average ± SEM from three independent experiments. ****p < 0.0001 and ####p < 0.0001 as described in STAR Methods. (D) Box and whisker plots show tumor volumes of T47D xenograft tumors grown with (+E2) or without E2 supplementation (−E2). Boxes depict interquartile range, center line represents median, and whiskers extend to minimum and maximum values for each group (n = 6). p values show significance comparing YFP −E2 to all other groups. See also Figure S2.

Of the eight identified *ESR1* fusions from all datasets that were PCR validated (Figure S1), only three in-frame fusions, ESR1-e6>YAP1 and ESR1-e6>PCDH11X from advanced disease and ESR1-e6>NOP2 from a primary tumor that showed subsequent resistance to endocrine therapy, produced stable proteins when expressed as cDNA, allowing further study (Figure 1B). Expression of all three fusion partner genes were highly expressed in patient tumors, as shown by expression rank plots for *YAP1*, *PCDH11X*, and *NOP2* translocation-bearing tumors relative to the expression of these genes among TCGA breast samples (Figure 1C). Relative RNA levels of transcripts were analyzed for each fusion partner, which showed increases in transcript levels beyond the fusion breakpoint for each gene examined, confirming that the fusion partner was disproportionately expressed versus the non-translocated allele (Figure 2A).

### In-Frame *ESR1* Fusions from Endocrine-Refractory Disease Confer Estrogen-Independent and Fulvestrant-Resistant Growth of ER^+^ Breast Cancer Cells

To test whether examples of *ESR1* in-frame gene fusions were drivers of endocrine therapy resistance, each fusion was individually expressed in two ER^+^ breast cancer cell line models: T47D and MCF7. Expression of fusion ER proteins in T47D cells was similar or lower than that observed in the WHIM18 PDX bearing the ESR1-e6>YAP1 fusion, indicating that phenotypic conclusions are not based on excess expression (Figure 2B). In addition, several out-of-frame *CCDC170* and an *AKR1D1* fusion event identified in this study (Table S1) were also engineered into T47D cells. Growth of *ESR1* fusion-expressing T47D was monitored in estradiol (E2)-deprived media and after addition of E2. Both in-frame fusions from advanced disease, ESR1-e6>YAP1 and ESR1-e6>PCDH11X, promoted estrogen-independent growth (Figure 2C, −E2), but the primary tumor fusion event, ESR1-e6>NOP2, had no growth-promoting properties. The out-of-frame events tested were also inactive (Figure S2A). E2 could stimulate growth in all conditions of fusion construct expression (Figure 2C, compare +E2 and −E2), suggesting that neither the *ESR1* in-frame active fusions (ESR1-e6>YAP1 and ESR1-e6>PCDH11X) nor the ESR1-e6 truncation, and not even the in-frame but inactive ESR1-e6>NOP2 fusion, could function as a dominant-negative on endogenous ER. Cells were treated with fulvestrant to degrade endogenous ER, while retaining expression of intact *ESR1* fusions that cannot bind drug or ligand, to test the specific contribution of the fusions to E2-independent growth. As expected, endogenous ER was degraded by fulvestrant, whereas levels of *ESR1* fusion proteins, as well as an ESR1-e6 truncation construct, were unaffected (Figure S2B), and growth promoted by ESR1-e6>YAP1 and ESR1-e6>PCDH11X was resistant to fulvestrant treatment (Figure 2C, −E2, +Fulvestrant). There was lack of additional growth promotion by the fusions when E2 was added in the presence of fulvestrant (Figure 2C, compare +E2, +Fulvestrant and −E2, +Fulvestrant). However, under these same conditions (Figure 2C, +E2, +Fulvestrant), growth induced by the *YAP1* and *PCDH11X* fusions remains significantly greater than controls (YFP and ESR1-WT [wild-type]). These results were confirmed in a second ER^+^ breast cancer cell line, MCF7 (Figures S2C-S2D). The *NOP2* fusion was highly expressed in the MCF7 cell line, in contrast to *NOP2* fusion-expressing T47D, but still lacked growth-promoting activity in hormone-deprived conditions, confirming that absence of functional effects was not due to inadequate expression of the *NOP2* fusion.

The ability of the three ESR1-e6-containing in-frame fusions to induce estrogen-independent growth was further tested *in vivo* in a xenograft study with stable T47D cells without supplementary E2. As controls, T47D YFP cells were used with supplementary E2. Results showed that control YFP −E2 cells produced negligible tumor growth compared with YFP cells +E2 (Figure 2D). However, T47D cells expressing *YAP1* and *PCDH11X* in-frame *ESR1* fusions formed tumors significantly larger than YFP −E2, while the cells expressing the *NOP2* fusion did not (Figure 2D).

### Active *ESR1* Fusions Promote Estrogen-Independent Gene Expression

**Figure 3.**
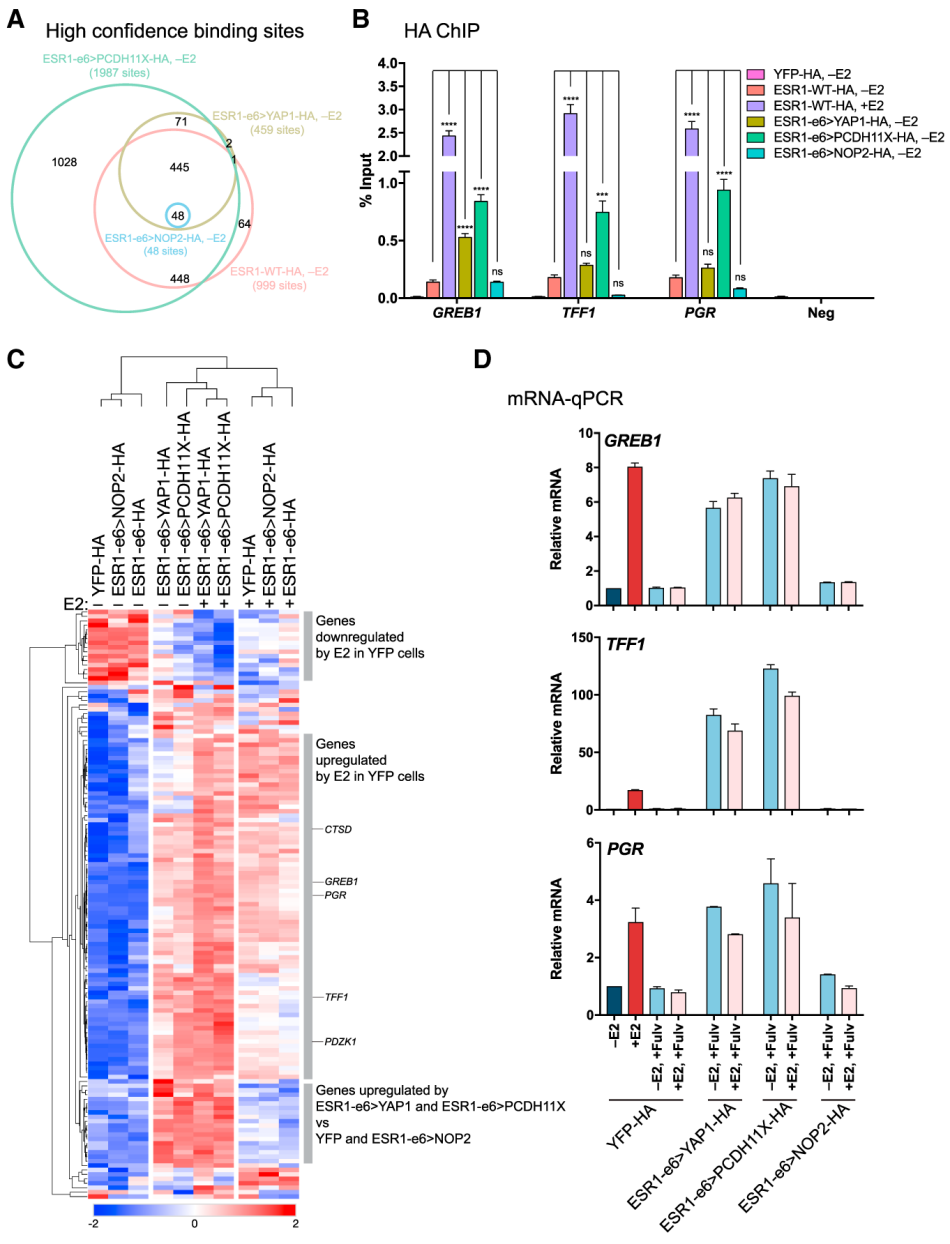
Active *ESR1* Fusions Promote Estrogen-Independent Expression of Target Genes (A) Venn diagram depicting overlap of binding sites from hormone-deprived stable T47D cells expressing HA-tagged *ESR1* constructs identified by HA-ChIP-seq. (B) HA-ChIP followed by qPCR for ER-binding regions of ER-responsive genes and negative ER-binding region. Bar graphs show average values from three experiments ± SEM. Asterisks denote significant differences as described in STAR Methods. (C) Heatmap showing differentially expressed genes near 445 sites bound by ESR1-e6>YAP1, ESR1-e6>PCDH11X, and ESR1-WT identified in (A). Known ER-responsive genes are indicated (*CTSD, GREB1, PGR, TFF1*, and *PDZK1*). Scale bar indicates row *Z* score. (D) Bar graphs depicting relative fold changes of estrogen-responsive genes whose ER-binding regions were examined in (B) from hormone-deprived stable T47D cells, normalized to YFP −E2 (dark blue bar), after E2 addition (+E2, red bar), or in combination with fulvestrant (light blue and pink bars). −E2 and +E2 for *ESR1* fusion-expressing cells have been omitted for clarity; see Figure S3F for complete data. Data are shown as averages from two independent experiments ± SEM. See also Figure S3.

To explore transcriptional properties associated with the *ESR1* fusion proteins described above, genome-wide binding of HA-tagged *ESR1* fusions was examined by HA chromatin immunoprecipitation followed by next-generation sequencing (ChIP-seq) in hormone-deprived stable T47D. ChIP-seq identified 445 binding regions shared by ESR1-WT, ESR1-e6>YAP1, and ESR1-e6>PCDH11X (Figure 3A). Very few sites were bound by ESR1-e6>NOP2 fusion despite high expression of HA-tagged *NOP2* fusion (Figure S3E), supporting earlier observations of inactivity in functional studies (Figures 2C, 2D, and S2C). ChIP-qPCR confirmed recruitment of ER to regulatory regions of known estrogen-responsive genes in a ligand-dependent manner in cells expressing WT-ER (Figure 3B). Additionally, both *YAP1* and *PCDH11X* fusions showed estrogen-independent enrichment at regulatory regions of established estrogen-responsive genes. For example, both fusions were enriched at the promoter of a canonical ER-regulated gene, *GREB1*, and the *PDCH11X* fusion was also enriched at enhancer estrogen response elements (EREs) of *TFF1* and *PGR* (Figure 3B).

To investigate whether expression from genes bound by *ESR1* fusions was modulated, RNA-seq was performed. Hierarchical clustering was conducted on differentially expressed genes near 445 shared sites bound by ESR1-WT, *YAP1*, and *PCDH11X* fusions, as indicated by the ChIP-seq data (Figure 3C). Upon stimulation with E2, the expression pattern of YFP control cells clustered away from unstimulated YFP cells, with enrichment for differential expression of estrogen-responsive genes. The *YAP1* and *PCDH11X* fusion-expressing cells had expression patterns that clustered together under estrogen-deprived and stimulated conditions and with E2-stimulated YFP cells. The transcriptionally active *ESR1* fusions maintained expression of estrogen-regulated genes in low-estrogen conditions at levels observed in YFP control cells in the presence of E2, demonstrating strong estrogen-independent gene activation. mRNA-qPCR validation of *GREB1, TFF1*, and *PGR* expression confirmed estrogen-independent and fulvestrant-resistant gene regulation (Figures 3D and S3F), suggesting that the active *ESR1* fusions drive endocrine resistance in a canonical manner through ERE-dependent activation. Moreover, the estrogen-independent activity of the *YAP1* and *PCDH11X* fusions was also independent of endogenous WT-ER, as transcriptional activity was maintained after cells were treated with fulvestrant to degrade endogenous ER. Thus, functionally important heterodimer formation between *ESR1* fusion protein and WT-ER is not likely. This conclusion was also supported by the lack of *ESR1* fusion association with WT-ER in a co-immunoprecipitation assay (Figure S3D). In contrast, the ESR1-e6 truncation mutant and *NOP2* fusion clustered together with YFP control cells displaying similar patterns of ligand-dependent ER gene expression, supporting our earlier observations that the *NOP2* fusion lacks ability to bind a large repertoire of EREs but whose inactivity is not due to mis-localization outside the nucleus, as staining for HA-tagged *ESR1* fusions constructs demonstrated nuclear localization (Figure S3A). These data were further supported by ERE-luciferase reporter experiments in HEK293T cells (Figure S3B). ESR1-WT drove estrogen-dependent expression of the ERE-luciferase reporter. In contrast, both ESR1-e6>YAP1 and ESR1-e6>PCDH11X as well as the ESR1-Y537S activating mutant drove estrogen-independent expression of the ERE-luciferase reporter. The level of activation by ESR1-e6>YAP1 was substantially higher than ESR1-e6> PCDH11X, which had activity intermediate to that achieved by the constitutively active ESR1-Y537S mutant and ESR1-e6>YAP1 (Li et al., 2013). In contrast to the ESR1-e6>YAP1 and ESR1-e6>PCDH11X fusions, neither the ESR1-e6 truncation mutation nor the ESR1-e6>NOP2 fusion drove expression of the ERE reporter. The transcriptional inactivity of the *NOP2* fusion was not due to abrogation of ERE binding, as pulldown experiments with a biotinylated concatenated ERE probe with a mutant ERE as a control demonstrated sequence-specific binding for all in-frame fusions (Figure S3C). In summary, our observations suggest that the inactivity of the *NOP2* fusion may be due to a failure to access chromatin in the nucleus of intact cells, rather than an inability to bind DNA per se.

### Active *ESR1* Fusions Promote Metastasis by Upregulating an EMT-like Transcriptional Program

**Figure 4.**
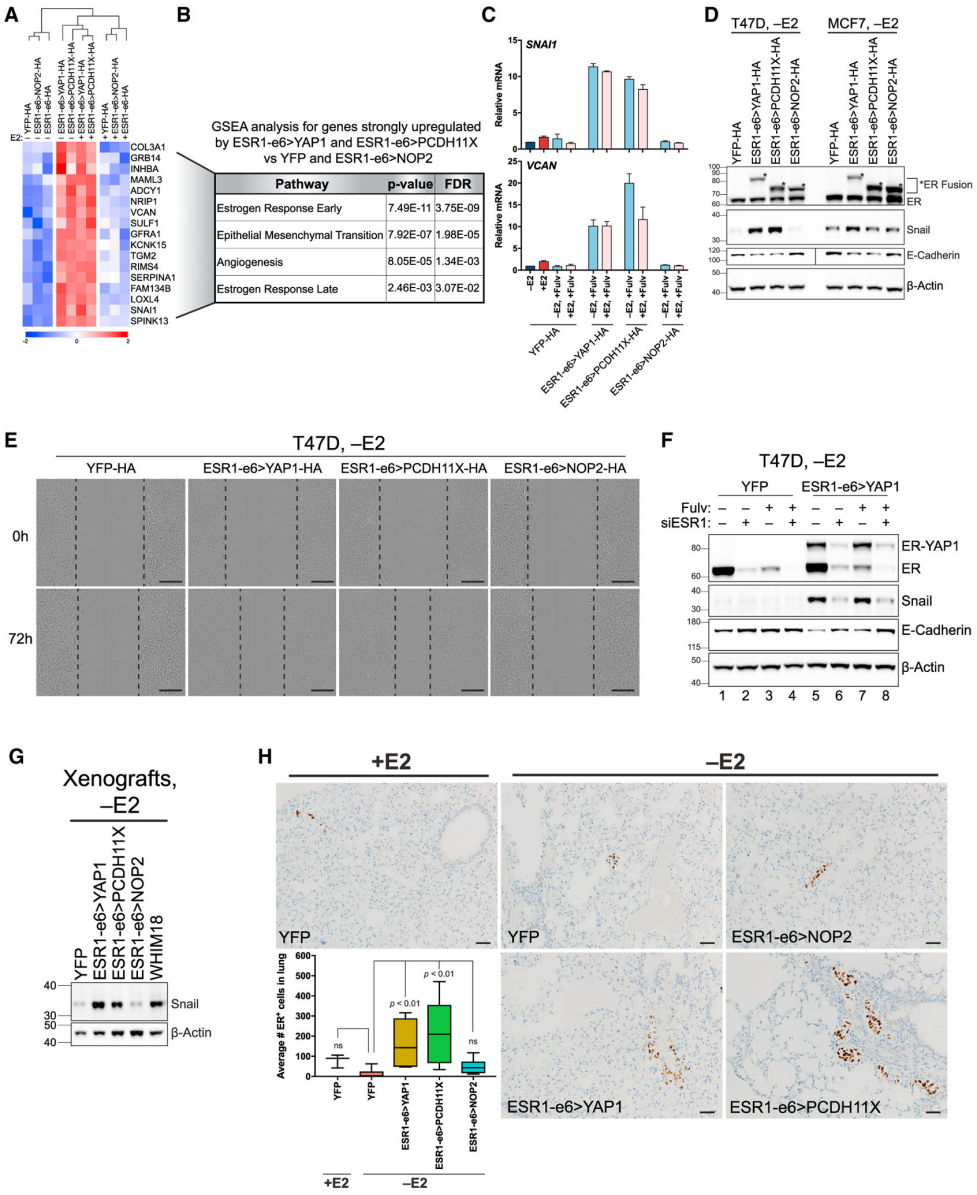
Active *ESR1* Fusions Promote Metastasis by Upregulating an EMT-like Transcriptional Program (A) Heatmap depicting genes upregulated by ESR1-e6>YAP1 and ESR1-e6>PCDH11X versus YFP and ESR1-e6>NOP2 (from bottom of Figure 3C). Scale bar indicates row *Z* score. (B) GSEA using genes identified in (A). (C) Bar graphs depicting expression of *SNAI1* and *VCAN*, by mRNA-qPCR in hormone-deprived stable T47D cells (−E2). Values are normalized to YFP −E2 (dark blue bar), treated with E2 (+E2, red bar), and in combination with fulvestrant (light blue and pink bars). −E2 and +E2 conditions for all cell lines are shown in Figure S4B. Data are averages of two independent experiments ± SEM. (D) Immunoblotting for endogenous ER (ER) and ER fusion (asterisks) using an N-terminal ERα antibody, Snail, and E-cadherin in hormone-deprived stable T47D and MCF7 cells. Vertical line in E-cadherin blot indicates different exposures taken for T47D and MCF7. (E) Scratch wound healing assay images of hormone-deprived stable T47D at 0 and 72 hr post-wounding. Dotted black line indicates leading edge of cells. Scale bar, 300 μm. (F) Immunoblotting of hormone-deprived T47D cells pre-treated with vehicle (Fulv−) or fulvestrant (Fulv+) before transfecting negative control siRNA (siRNA−) or siRNA against the N terminus of *ESR1* (siESR1+). (G) Immunoblotting for Snail in T47D xenograft and WHIM18 PDX tumors. (H) ER IHC images performed on lungs of mice bearing T47D xenografted tumors from Figure 2D. Box and whiskers plots show IHC quantification of ER+ cells, with boxes depicting interquartile range, center line representing median value, and whiskers extending to minimum and maximum values for each group (n=5). p values indicate significance comparing YFP −E2 versus fusion-bearing groups or versus YFP +E2. Scale bar, 100 μm. See also Figure S4 and Table S3.

A cluster of genes was identified that was selectively upregulated by the active *YAP1* and *PCDH11X* fusions (Figures 3C and4A). Gene set enrichment analysis (GSEA) was used to examine pathway enrichment in this cluster, which indicated significant enrichment of estrogen response pathways as well as an epithelial-to-mesenchymal transition (EMT)-like signature (Figure 4B). The EMT signature included *TGM2, COL3A1, INHBA*, and *VCAN*. One of the best-described EMT genes, *SNAI1*, was also selectively upregulated by both active fusions. Analysis of binding site distances to transcription start sites (TSSs) of genes in this cluster demonstrated that the majority of binding occurs at distances >50 kb from the TSS (Table S3). This suggests a propensity of the active *YAP1* and *PCDH11X* fusions to bind in enhancer regions upstream and downstream of these genes, characteristic of the ER cistrome reported in the literature (Carroll et al., 2006). Motif analysis of these binding sites showed enrichment for the ERE motif (Figure S4A), suggesting that the direct regulation of EMT genes by the active *YAP1* and *PCDH11X* fusions is mediated by enhancer and more distant range interactions. Upregulation of *VCAN* and *SNAI1* transcripts (Figures 4C and S4B) and Snail protein (Figure 4D) was orthogonally validated. In MCF7 cells, whose basal levels of Snail were higher in YFP controls compared with T47D YFP, showed an induction of Snail by ESR1-e6>YAP1, but not by ESR1-e6>PCDH11X, suggesting a degree of cell context-dependent effects (Figure 4D). Upregulation of Snail protein was also confirmed in T47D xenograft tumors and in a PDX model naturally harboring the ESR1-e6>YAP1 fusion (WHIM18) (Figure 4G). Expression of *SNAI1* was unaffected by fulvestrant treatment in T47D cells, consistent with the conclusion that upregulation of EMT genes by the active fusions is independent of endogenous WT-ER (Figures 4C and S4B).

ChIP-seq also identified 71 selectively bound sites by ESR1-e6>YAP1 and ESR1-e6>PCDH11X not bound by ESR1-WT nor ESR1-e6>NOP2 (Figure S4C). GSEA pathway analysis of differentially expressed genes near these sites showed enrichment for UV radiation response genes, as well as enrichment for EMT genes, with *TGFBR3* and *GJA1* contributing to EMT pathway enrichment (Figure S4C). *TGFBR3* encodes for transforming growth factor-β receptor III and has roles in migration and invasion (Gatza et al., 2010). *GJA1* encodes for connexin-43, a gap junction protein whose expression in breast cancer cells has been implicated in pulmonary metastasis (Elzarrad et al., 2008), consistent with observed lung metastasis in both patients from which the ESR1-e6>YAP1 and ESR1-e6>PCDH11X fusions were identified.

A decrease in E-cadherin levels from *YAP1* and *PCDH11X* fusion-expressing cells was observed relative to YFP control and *NOP2* fusion-expressing cells (Figure 4D), and a decrease in cell surface E-cadherin was also observed, consistent with an EMT-like transition (Figures S4E and S4F). However, there was no detectable increase in vimentin levels, suggesting that the *YAP1* and *PCDH11X* fusions drive a partial EMT gene expression pattern that nonetheless can be metastasis associated (Jolly etal., 2015). To examine the functional consequences of the active fusions with respect to the metastatic process, cell motility was examined. The *YAP1* and *PCDH11X* fusions induced significantly greater wound recovery and motility than YFP controls and *NOP2* fusion-expressing cells (Figure 4E, quantified in Figure S4D). To exclude the possibility that EMT-associated gene expression was due to phenotypic drift of cells under long-term selection, small interfering RNA (siRNA)-mediated knockdown of ESR1-e6>YAP1 fusion was examined to determine whether EMT-associated features could be reversed. Estrogen-deprived stable T47D YFP control or ESR1-e6>YAP1-expressing cells were pre-treated with fulvestrant to degrade endogenous WT-ER, before transfecting with negative control siRNA (siESR1–) or siESR1 against the N terminus of *ESR1* (siESR1+). Forty-eight hours post-transfection, Snail protein levels were markedly reduced in ESR1-e6>YAP1 cells after siESR1 transfection with or without fulvestrant pre-treatment compared with siESR1 – with or without fulvestrant (Figure 4F, compare lanes 5 and 7 with lanes 6 and 8). In addition, cells with decreased Snail as a result of ER-YAP1 fusion protein knockdown tended to have higher levels of E-cadherin, suggesting that knockdown of the ESR1-e6>YAP1 fusion transcript restores these aspects of a typical epithelial gene expression pattern. Similar effects were confirmed in stable MCF7 cells expressing ESR1-e6>YAP1 (Figure S4G, compare lanes 5 and 7 with lanes 6 and 8), although Snail levels were more affected by fulvestrant pre-treatment alone, showing that higher basal levels of Snail in MCF7 cells can also be driven by WT ESR1 (Figure S4G, compare lanes 1 and 3 for YFP-expressing cells and lanes 5 and 7 for ESR1-e6>YAP1-expressing cells). However, Snail expression is resistant to fulvestrant suppression in the presence of the ESR1-e6>YAP1 fusion (Figure S4G, compare lanes 3 and 7). The metastatic potential of fusion-expressing cells *in vivo* was measured by ER immunohistochemistry from the lungs, liver, and bones of mice bearing T47D xenografts from Figure 2D. The number of micrometastatic ER^+^ cells in the lungs of *YAP1* and *PCDH11X* fusion bearing mice was significantly greater than that in the lungs of mice bearing tumors generated from YFP control cells upon estrogen deprivation (Figure 4H). YFP control tumors grown with E2 supplementation were much larger (Figure 2D), but pulmonary micrometastasis was not significantly different from YFP controls −E2, demonstrating that differences in pulmonary metastasis potential associated with the active fusions were not due simply to differences in disease burden. Bone and hepatic micrometastases were not observed. Pulmonary metastasis in this model was not a feature of YFP control cells, even when disease burden was increased markedly with E2 supplementation. Taken together, these results suggest a role for active *YAP1* and *PCDH11X* fusions in driving pulmonary metastasis in association with the expression of genes known to contribute to EMT biology and metastatic behavior.

### Growth Driven by *ESR1* Fusions Can Be Suppressed with CDK4/6 Inhibitor Treatment

**Figure 5.**
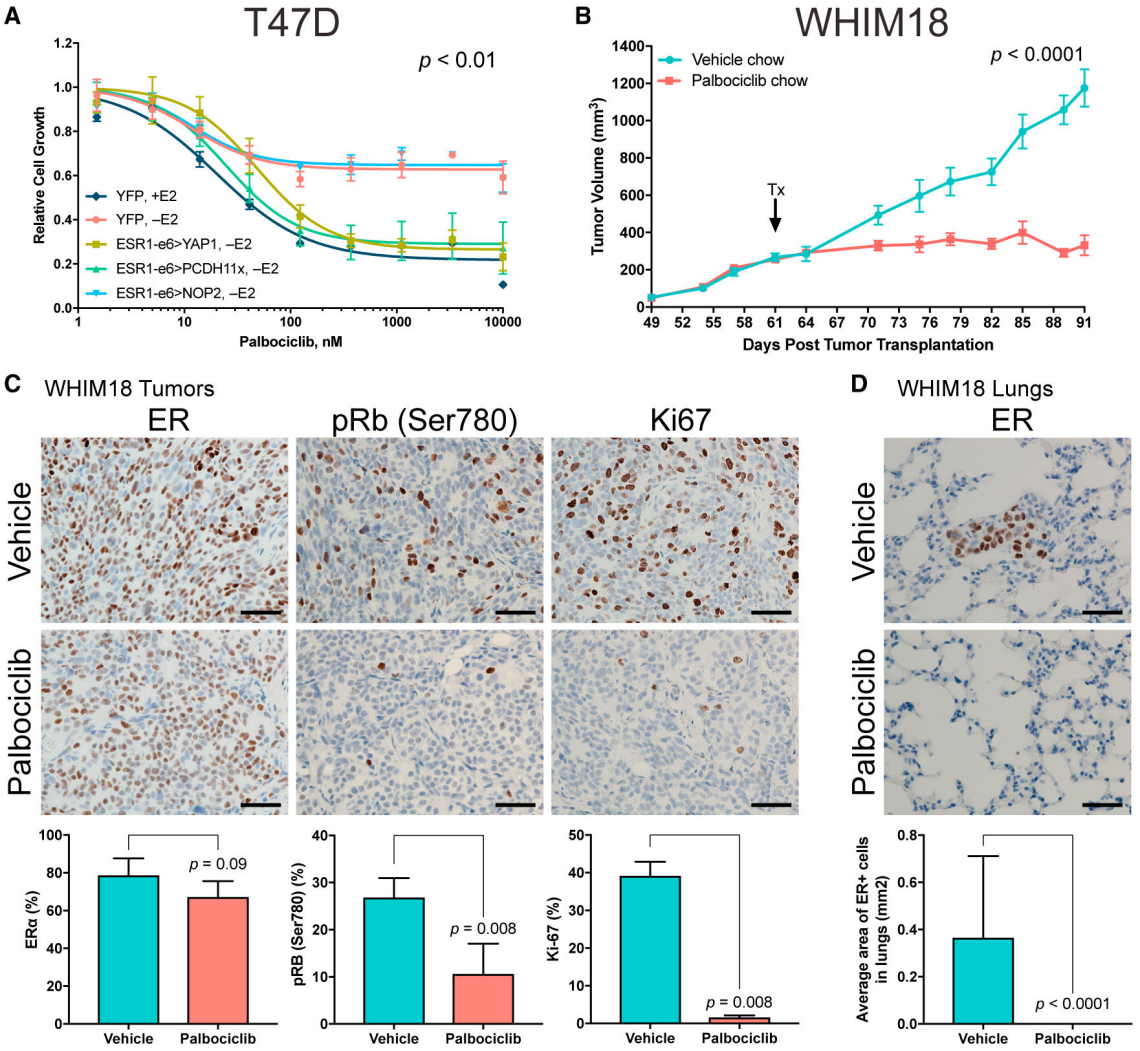
Growth Driven by *ESR1* Fusions Can Be Suppressed with CDK4/6 Inhibitor Treatment (A) Growth of hormone-deprived stable T47D cells in response to increasing concentrations of a CDK4/6 inhibitor, palbociclib. YFP +E2 used as control. P value describes significance between YFP +E2, ESR1-e6>YAP1, and ESR1-e6>PCDH11x slopes compared with YFP −E2. Data shown are averages of three independent experiments ± SEM. (B) Tumor volumes of WHIM18 PDX in the absence of exogenous E2 supplementation. Arrow indicates treatment start (Tx) with vehicle or palbociclib containing chow. P value describes significance of tumor growth rates (slopes) derived from tumor volumes at day of randomization to experiment end. Data are shown as averages from 8–11 mice per treatment group ± SEM. (C) Representative IHC images for ER, pRb, and Ki-67 from vehicle and palbociclib-treated WHIM18 tumors. Quantification of IHC staining below with significance comparing treatment groups. Data are averages counts from five tumor sections from each treatment group, with error bars representing SD. (D) ER IHC images of lungs from WHIM18-bearing mice. Micrometastatic ER^+^ lesions were quantified by measuring area of ER^+^ cells. Data are shown as average ER^+^ areas from five lung sections per treatment group. P value determined as in (C). Scale bar, 100 μm in (C) and (D). See also Figure S5.

The loss of the LBD renders the function of *ESR1* fusion genes resistant to all endocrine treatments, and therefore alternative therapies will be necessary to treat patients who present with active *ESR1* fusions. Palbociclib, a selective CDK4/6 inhibitor was chosen for study because of our recent report that this agent can antagonize the growth of tumors expressing *ESR1* mutations as long as phospho-Rb (pRb) is present (Wardell et al., 2015). Because the target of activated CDK4/6 is Rb, pRb levels were examined by immunohistochemistry (IHC) in *ESR1* fusion-expressing T47D xenograft tumor sections (Figure S5A). pRb levels in *YAP1* and *PCDH11X* fusion xenograft tumors grown without E2 supplementation were comparable with YFP controls +E2 and were elevated relative to YFP −E2 and NOP2 fusion-containing tumors. T47D stable cells expressing YFP and the three in-frame *ESR1* fusions were treated with palbociclib under hormone-deprived conditions and growth-inhibitory effects were assessed (Figure 5A). Palbociclib inhibited T47D cell growth driven by the *YAP1* and *PCDH11X* fusions in a dose-dependent manner. A similar palbociclib effect was observed in *ESR1* fusion-expressing MCF7 stable cells (Figure S5B). To test palbociclib sensitivity *in vivo*, a PDX model naturally harboring the ESR1-e6>YAP1 fusion (WHIM18) was exposed to palbociclib. Consistent with *in vitro* results, tumor growth in the PDX model was inhibited in mice treated with palbociclib compared with vehicle-treated mice (Figure 5B; tumor growth rates shown in Figure S5C). Palbociclib-treated WHIM18 tumors also showed significant reduction in pRb and marked decrease in Ki-67 levels, without altering levels of ER (Figure 5C) or progesterone receptor (PR) (Figure S5D). Areas containing micrometastatic ER^+^ cells observed in the lungs of vehicle chow-treated WHIM18 mice were not seen in palbociclib-treated mice (Figure 5D), suggesting that pulmonary metastatic frequency could also be downregulated by CDK4/6 inhibition.

## DISCUSSION

This study demonstrated that two in-frame *ESR1* fusions in a small late-stage cohort of metastatic ER^+^ cases drive not only endocrine therapy resistance but also metastatic disease progression. The functional characterization of *ESR1* fusions’ properties described herein should drive efforts to identify and further characterize additional *ESR1* fusions in early- and late-stage ER^+^ breast cancer.

The ability to block active *ESR1* fusion-induced growth with a CDK4/6 inhibitor has important implications for clinical practice. Patients with active *ESR1* fusions may present with a clinical pattern of rapidly progressing disease despite adjuvant or metastatic endocrine therapy treatment and therefore be offered chemotherapy instead of a CDK4/6 inhibitor-containing regimen. Because therapeutically resistant disease is infrequently re-biopsied and even more rarely analyzed using RNA-seq, a prospective study of *ESR1* in-frame fusion-expressing ER^+^ tumors will be required to establish an effective approach for these tumors.

Although *ESR1* fusions are challenging to diagnose because of variable 3′ fusion partners, evidence for additional *ESR1* fusions is emerging in the literature. For example, ESR1-e6>DAB2 and ESR1-e6>GYG1 were both identified in metastatic ER^+^ breast cancer (Hartmaier et al., 2018). Like the active *ESR1* fusions we describe herein, ESR1-e6>DAB2 and ESR1-e6>GYG1 follow the same pattern (i.e., *ESR1* exon 6 in-frame fusions with 3′ partners provided by inter-chromosomal translocation). Thus, this type of *ESR1* fusion gene structure is most clearly linked to endocrine therapy resistance. Several precision medicine programs now include RNA-seq in their standard pipelines, and thus much more data on ESR1-e6 in-frame fusion prevalence should be available soon.

Because active *ESR1* fusions induce pRb (Figure S5A), pRb might also be an appropriate marker to guide CDK4/6 inhibitor therapy and might provide strong pre-clinical rationale to potentially examine pRb levels in patients on Als to define populations for CDK4/6 inhibition. This idea is supported by our previous report, in which the growth of endocrine-refractory PDX tumors remained sensitive to CDK4/6 inhibition, as long as those tumors express pRb under estrogen-deprived growth conditions (Wardell etal., 2015).

The inactivity of the ESR1-e6>NOP2 fusion is surprising, as the expressed recombinant protein is stable. This demonstrates that not every in-frame ESR1-e6 fusion is active with respect to endocrine therapy resistance. The *NOP2* fusion may have other biological properties that we were unable to detect in our experimental model systems. The out-of-frame *ESR1* fusions also had no growth-promoting properties but could also be active though novel mechanisms.

The role of active *ESR1* fusions in promoting EMT-like gene expression changes follows a pattern associated with other members from a diverse family of cancer-associated gene fusion events. For example, the TMPRSS2-ERG fusion in prostate cancer has also been reported to directly regulate cell migration genes (Tian et al., 2014). Given the diverse structures of EMT-inducing *ESR1* fusions revealed here with the study of just two, it is also possible that more EMT and motility-inducing transcription factor gene fusions remain to be discovered, and the formation of these could be primary drivers of metastasis.

## STAR★METHODS

### CONTACT FOR REAGENT AND RESOURCE SHARING

Further information and requests for resources and reagents should be directed and will be fulfilled by the Lead Contact Matthew J. Ellis (mjellis@bcm.edu).

## EXPERIMENTAL MODEL AND SUBJECT DETAILS

### Cell Lines

All cell lines were purchased from ATCC and cultured at 37°C in 5% CO_2_. All cell lines were authenticated and tested for mycoplasma. HEK293T and MDA-MB-231 cells were cultured in DMEM with L-Glutamine and 4.5 g/L glucose (HyClone) supplemented with 10% FBS (cat# F0926, Sigma) and 1% penicillin-streptomycin (Invitrogen). T47D and MCF7 cells were cultured in RPMI1640 with L-Glutamine (Mediatech) supplemented with 10% FBS, glucose to 4.5 g/L (Sigma), 10 mM HEPES (GenDEPOT), 1 mM sodium pyruvate (GenDEPOT), and 50 μg/mL gentamycin (GenDEPOT). Estrogen/hormone deprivation was performed by plating cells in culturing media overnight followed by washing with PBS and replacing with hormone deprived media consistaing of phenol red free media supplemented as described above but with 10% charcoal-stripped serum (CSS) (cat# F6765, Sigma), followed by changing with hormone-deprived media every 2-3 days for 5-7 days.

### *In Vivo* Animal Studies

All animal experiments were carried out in strict accordance with the guidelines recommended for care and use of laboratory animals by the National Institutes of Health. The Animal Studies Committee at Washington University (St. Louis, MO, USA) approved all animal protocols used for T47D xenograft studies. Three-week old NOD/SCID gamma female mice were purchased from Jackson Laboratories. Stable T47D cells were trypsinized, counted, washed by PBS, and suspended in ice cold serum free RPMI medium at 10 × 10^6^ cells per 100 μL. Matrigel was added to a final 33% by volume. 150 μL mix (10×10^6^ cells) was injected subcutaneously into the mouse flanks bilaterally. Six mice were injected per group. Tumor volumes were measured by caliper weekly. For PDX studies, all animal procedures were approved by the Institutional Animal Care and Use Committee at Baylor College of Medicine (Houston, TX, USA) (protocol# AN-6934). 2-3 mm tumor pieces from a second generation growing WHIM18 tumor were engrafted into cleared mammary fat pads of 3-4 weeks old SCID/bg mice (Charles River) and allowed to grow without exogenous E2 supplementation until tumors reached 150-400 mm^3^. Mice were then randomized to receive vehicle or palbociclib (Pfzier) containing chow (daily dose of 70mg/kg per day) for an additional 30 days (11 mice per group). Tumor volumes were measured by caliper every 3-4 days. For all animal experiments, tumor volumes were calculated by V = 4/3 × π × (length/2)^2^ × (width/2). Animals were sacrificed when tumors reached 1500 mm^3^ or at the study end time point. Tumors and organs were harvested and frozen in liquid nitrogen for storage or fixed in 4% formaldehyde overnight at RT, then held in 70% ethanol before paraffin embedding, sectioning (5 μm) and subsequent IHC processing.

### Clinical Samples

The primary breast cancer samples for this study were either accrued from two neoadjuvant endocrine therapy trials (Ellis et al., 2011; Olson et al., 2009) or analyzed from TCGA breast samples (Ciriello et al., 2015). The methodologies for RNA extraction and expression profiling experiments have been previously published (Ellis et al., 2011). Frozen metastatic biopsy samples from patients with advanced breast cancer (Table S2) were accrued under a banking protocol approved by the Washington University School of Medicine Institutional Review Board (approval number 201102244).

## METHOD DETAILS

### *ESR1* Fusion Discovery Using ChimeraScan and INTEGRATE

Fusion candidates were discovered using ChimeraScan (Iyer et al., 2011) and INTEGRATE (Zhang et al., 2016) when whole genome sequencing data were available from 38 cases previously reported (Ellis et al., 2012). The Illumina RNA-Seq paired-end reads in FASTQ format were provided to ChimeraScan version 0.4.5, which was run using default parameters. The alignments (BAM format by TopHat2) of the RNA-seq reads are provided to INTEGRATE version 0.1, which is run using default parameters in RNA only mode. All the analysis was based on hg19. ChimeraScan results (bedpe format) are filtered by removing records with types marked as read through, overlapping converging, overlapping diverging, adjacent converging, and adjacent diverging. These could be transcriptome only variations or chimeras reported because of certain annotation issues. The gene fusions with *ESR1* gene as a fusion partner are picked out from all the fusion candidates discovered by the methods described above and from analysis done by TCGA.

### Molecular Cloning to Generate *ESR1* Fusion Constructs

cDNAs encoding ESR1-e6>NOP2, ESR1-e6>PCDH11X, and ESR1-e6>AKR1D1 were synthesized from patient RNAs via oligo-dT reverse transcription (RT) followed by polymerase chain reaction (PCR) using primers complementary to the 5′ and 3′ ends of the fusion genes. ESR1-e7>POLH and ESR1-e6>AKAP12 were generated from cDNAs encoding *ESR1, POLH*, and *AKAP12* by overlapping PCR extension/amplification as previously described for ESR1-e6>YAP1 (Li et al., 2013). All other constructs were created by standard PCR using pre-existing cDNA templates. Amplified DNA fragments were inserted into the lentiviral vector pFLRu-FH as described previously (Li et al., 2013). ESR1-e6>AKAP12 was generated but due to its exceptionally large size, could not be cloned into the lentiviral vector and subsequently proved hard to express upon transfection and was not studied further. Carboxy-terminal HA-tagged *ESR1* fusion constructs were generated by subcloning each construct from pFLRu-FH using primers for PCR that included BamHI and EcoRI restriction sites along with the HA sequence (STAR METHODS) into pCDH-CMV-MCS-EF1α-RFP-Puro vector (System Biosciences). All constructs in their final vectors were confirmed by Sanger sequencing.

### Lentiviral Production and Stable Cell Line Generation

Lentiviral production was performed as described previously (Li et al., 2013). Briefly, *ESR1* constructs cloned in pFLRu-FH and HA-tagged *ESR1* constructs in pCDH-CMV-MCS-EF1α-RFP-Puro (System Biosciences) and pCDH-CMV-MCS-ER1α-Puro (System Biosciences) vector DNAs were co-transfected with the packaging plasmids into HEK293T cells using Fugene HD (Roche). Culture media containing viruses were harvested after 48 hr, filtered, and added to T47D and MCF7 cells in the presence of polybrene. Stably infected cells were selected by 2 μg/mL puromycin (Sigma) two days after infection. Three sets of T47D stable cell lines were generated, one set expressing non-HA-tagged *ESR1* constructs (used in Figures 2, S2, 4G, 4H, 5A, and S5A), one set expressing HA-tagged *ESR1* constructs in pCDH-CMV-MCS-EF1α-RFP-Puro (used in Figures 3, S3D-S3F, 4A-4F, and S4A-S4F) and one set expressing HA-tagged *ESR1* constructs in pCDH-CMV-MCS-ER1α-Puro used in Figure S3A. Two sets of MCF7 cells were generated, one set expressing non-HA-tagged *ESR1* constructs (used in Figures S2C, S2D, and S5B) and HA-tagged ESR1 constructs in pCDH-CMV-MCS-ER1α-Puro (used in Figures 4D and S4G).

### *In Vitro* Growth Assays

Hormone independent cell growth was subsequently measured by low density triplicate plating of T47D or MCF7 cell lines in hormone-deprived media in 96-well plates (2000 cells/well) in the absence or presence of 10 nM E2 (Sigma) in combination without or with 10 nM fulvestrant (Selleckchem). Cell growth was quantified by Alamarblue assay at Day 1 and Day 12 post plating and relative growth was calculated as Day 12/Day 1 ratios. Remaining cells not used in the Alamarblue assay were plated in CSS containing media and grown further for 72h in the absence or presence of 10 nM fulvestrant before harvesting and subsequent processing for immunoblot analysis. For palbociclib sensitivity assays, T47D and MCF7 cells were hormone deprived for seven days, then plated in 96-well plates as described above in the absence of presence of 3-fold dilutions of palbociclib (cat# S1116, Selleckchem) from 10 μM down to 0.0015 μM for 12 days, changing hormone-deprived media and palbociclib every 2-3 days. Cell growth was quantified similarly as above and relative growth was calculated by taking the palbociclib treated Day 12/Day 1 ratio divided by the vehicle treated Day 12/Day 1 ratio.

### siRNA Knockdown

Stable T47D or MCF7 were hormone-deprived for 7-9 days before pre-treatment with DMSO vehicle or 1 μM fulvestrant for 24h prior to reverse transfection with RNAiMAX (Invitrogen) and 50 nM siRNA Universal Negative Control #1 (cat# SIC001, Sigma) or 50 nM siESR1 targeting N-terminal sequences of *ESR1* (Sigma). Fresh DMSO or 1 μM fulvestrant was added during the transfection. 48h post transfection, cells were collected by scraping and subjected to immunoblotting.

### Immunoprecipitation and Immunoblot Analysis

For IP assays, hormone deprived stable T47D cells were left untreated or stimulated with 10 nM E2 for 15’ at 37°C. Cells were harvested then lysed in IP lysis buffer [0.5% NP-40, 10% glycerol, 280 mM NaCl, 50 mM Tris pH 8.0, 2 mM EGTA, 0.2 mM EDTA, 1 mM PMSF, 1 mM sodium orthovanadate, 1 mM DTT, 1 μg/mL pepstatin, phosSTOP phosphatase inhibitor tablet (Roche), and complete EDTA-free protease inhibitor tablet (Roche)] for 20 min. 0.5 mg of clarified lysates were immunoprecipitated with an Anti-HA antibody (cat# 3724, Cell Signaling, 1:50) overnight at 4°C with rotation. Protein A magnetic beads (cat# 1614013, Bio-Rad) were added and rotated for 1h at 4°C followed by extensive washing with IP lysis buffer. Immunoprecipitated samples along with 25 μg of whole cell lysates (inputs) were heated at 90°C before loading onto SDS-PAGE gels (Invitrogen) and electroblotted onto nitrocellulose membranes (Bio-Rad). Whole cell lyates for all other immunoblotting procedures were prepared in RIPA buffer and blotted as described previously (Li et al., 2013). Fresh frozen WHIM18 tumors were cryopulverized (Covaris CP02) then lysed in RIPA buffer. The following primary antibodies were used for blotting: N-terminal estrogen receptor α (cat# 04-820, Millipore, 1:1000), C-terminal estrogen receptor α (cat# sc-543, Santa Cruz, 1:1000), E-Cadherin (cat#14472, Cell Signaling, 1:1000), and Snail (cat #3879, Cell Signaling, 1:500). β-Actin (cat# A5316, Sigma, 1:5000) used as loading control for all immunoblots.

### Dual Luciferase ERE Reporter Assay

To test ER fusion effect on wild-type ERE activation ability, 60 ng of empty pCDH-CMV-MCS-ER1α-Puro Vector, ESR1-WT-HA, ESR1-e6>YAP1-HA, ESR1-e6>PCDH11X-HA, ESR1-e6>NOP2-HA, ESR1-e6-HA, or ESR1-Y537S-HA were co-transfected by reverse transfection using Lipofectamine 2000 (Invitrogen) together with 25,000 hormone-deprived HEK293T cells per well in triplicates in a 96-well plate with 60 ng Firefly luciferase reporter vector (driven by three copies of vitellogenin Estrogen Response Element (11354, Addgene) (Hall and McDonnell, 1999) and 5 ng control Renilla luciferase vector (pGL4.70, Promega). Prior to transfection, HEK293T cells were cultured in hormone deprived media containing charcoal-stripped serum for seven days. One day after transfection, cells were either left unstimulated or stimulated with 2.5 nM E2 for 24h. On the following day, cells were quantified for the firefly and Renilla luciferase levels using the Dual-Glo Luciferase assay kit (Promega). Averages of Firefly/Renilla luminescence readings from each sample were calculated and expressed as fold change in activity relative to Vector transfected −E2.

### Biotinylated 3X ERE Pulldown

All 5′ biotinylated DNA were synthesized by Integrated DNA Technologies. The sequence of the wild-type and mutant 3XERE were GTAGGTCACTGTGACCTAGACGCAGGTCACTGTGACCTAGACGCAGGTCACTGTGACCGT and GTAGATCACTGTGAACTAGA CGCAGATCACTGTGAACTAGACGCAGATCACTGTGAACGT, respectively. Each DNA and its complement were annealed by boiling at 95°C for 15 min and allowed to cool overnight at room temperature. Each biotinylated DNA was bound to streptavidin M-280 Dynabeads (Invitrogen) per manufacturer’s directions and washed with NETN buffer (20 mM Tris, pH 8.0,150 mM NaCl, 0.5mM EDTA, and 0.5% NP-40) before incubation with 200-1000 μg of HEK293T extracts (whole cell or nuclear extracts) transiently transfected with the indicated expression constructs. Protein/DNA extracts were rotated at 4°C for 1h then washed four times with NETN buffer and analyzed by immunoblotting.

### ChIP-seq

#### Chromatin preparation

Stable T47D cells were hormone deprived for 7 days in charcoal-stripped containing media before fixing at 1% formaldehyde (cat# F8775, Sigma) while swirling for 10 min at RT. To quench, glycine was added to 0.2 M and incubated for another 5 min at RT. Cells were then washed and harvested in cold TBSE (20 mM Tris-HCl pH 7.5, 150 mM NaCl, 1 mM EDTA). After further washing in TBSE, cells were lysed in 0.1% SDS buffer (50 mM HEPES-KOH pH 7.5, 150 mM NaCl, 1 mM EDTA, 1% Triton X-100, 0.1% sodium deoxcholate, 0.1% SDS, cOmplete EDTA-free protease inhibitor cocktail tablet) for 15 min at 4°C with rotation. Samples were centrifuged and washed 3X with 0.1% SDS buffer and resultant nuclear pellets were lysed with 1% SDS buffer for 15 min at 4°C with rotation. After washing with 0.1% SDS buffer, nuclear lysates were centrifuged at 20,000 rpm and resultant chromatin pellets were resuspended in 0.1% SDS buffer with 0.5 mm glass beads (cat# 11079105, Biospec). The chromatin solution was sonicated with a Branson Sonifier S450D with 18, 30 s pulses at 40% amplitude. Crosslinks were reversed by incubating sonicated chromatin with pronase (Roche) at 42°C for 2h followed by incubation at 67°C for 6h. Phenol:chloroform (Ambion) extraction was used to isolate sonicated chromatin that contained DNA fragments 200-500 bp in size that was confirmed by agrose gel electrophoresis.

#### Chromatin immunoprecipitation

Dynabeads Protein G (ThermoFisher) were equilibrated in 0.1% SDS buffer then a portion was added to chromatin extracts from above to pre-clear for 2h at 4°C with rotation. HA antibody (cat# sc-7392, Santa Cruz) was added to the remaining Protein G and allowed to bind for 2h at 4°C with rotation. Pre-cleared chromatin extracts were then added to antibody-bound beads and rotated overnight at 4°C followed by extensive 5 min washes 0.1% SDS buffer, then once in 0.1% SDS buffer containing 0.35 M NaCl, then once in ChIP wash buffer (10 mM Tris-HCl pH 8.0, 250 mM LiCl, 1 mM EDTA, 0.5% NP-40, 0.5% sodium deoxycholate), then once in TE buffer. Elution was performed by pelleting and resuspending in ChIP buffer and heating at 68°C for 1h with agitation. Samples were pelleted, reuspended in TE buffer and crosslinks were reversed with pronase and heating at 42°C for 2h followed by incubation at 67°C overnight. Chromatin isolation was then performed using phenol:chloroform extraction and used for ChIP-qPCR and subsequently processed for next generation sequencing as follows:

#### Next generation sequencing

The Biopolymers Facility (Harvard Medical School, Boston, MA, USA) conducted quality control testing on an Angilent BioAnalyzer followed by Wafergen PrepX DNA ChIP library preparation. Pooled libraries were loaded onto two lanes of HiSeq Rapid v2 flow cell (Illumina) with PhiX control adaptor-ligated library (Illumina) spiked-in at 5% by weight to ensure balanced diversity and to monitor clustering and sequencing performance. Single-end 50 bp reads were generated on a HiSeq 2500 Sequencing System.

#### RNA-seq

Stable T47D cells were hormone deprived for 5 days in charcoal-stripped containing media then grown for another 48h in the absence or presence of 10 nM E2. RNA was isolated using RNeasy Mini Kit (QIAGEN) according to manufacturer’s directions and subjected to on column DNase (QIAGEN) digestion to remove genomic DNA before final elution in water. The Genomic and RNA Profiling Core (Baylor College of Medicine, Houston, TX, USA) conducted sample quality checks using the NanoDrop spectrophotometer and Agilent Bioanalyzer 2100 followed by subsequent Illumina TruSeq Stranded mRNA library preparation protocol (p/n 15031047, rev. E) as follows: A double-stranded DNA library was created using 180ng of total RNA (measured by picogreen), with the Illumina TruSeq Stranded mRNA-Seq Sample Prep kit (cat# RS-122-2101). First, cDNA was created using the fragmented 3′ poly(A) selected portion of total RNA and random primers. During second strand synthesis, dTTP is replaced with dUTP which quenches the second strand during amplification, thereby achieving strand specificity. Libraries were created from the cDNA by first blunt ending the fragments, attaching an adenosine to the 3′ end and finally ligating unique adapters to the ends. The ligated products were then amplified using 15 cycles of PCR. The resulting libraries were quantified using a NanoDrop spectrophotometer and fragment size assessed on an Agilent Bioanalyzer. A qPCR quantification was performed on the libraries to determine the concentration of adaptor ligated fragments using Applied Biosystems ViiA7 Real-Time PCR System and a KAPA Library Quant Kit (cat# KK4824).

Using the concentration from the ViiA7 qPCR machine above, 27 pM of library was loaded onto two lanes of a high output v4 flowcell (Illumina p/n PE-401-4001) and amplified by bridge amplification using the Illumina cBot machine (cBot protocol: PE_HiSeq_Cluster_Kit_v4_cBot_recipe_v9.0). PhiX Control v3 adaptor-ligated library (Illumina p/n 15017666) is spiked-in at 2% by weight to ensure balanced diversity and to monitor clustering and sequencing performance. Paired-end 100 bp reads were generated on a HiSeq 2500 Sequencing System (Illumina p/n FC-401-4003).

#### Quantitative PCR

qPCR was performed using SsoAdvanced SYBR green Supermix (Bio-Rad) and 0.5 mM primers (Sigma) listed in Key Resources Table and run on a LightCycler 96 (Roche). All samples were run in triplicate and values shown are the average ± SEM of at least 2 independent experiments. For ChIP-qPCR, 1% inputs were run for each corresponding sample and primers against a region on Chr20 which ERα does not bind was used as a negative control. Chromatin captured from HA-ChIP in YFP-HA cells were used as control instead of IgG antibody alone. For mRNA-qPCR (Figures 3D, S3F, 4C, and S4B), RNA was extracted as described above from stable T47D cells grown in hormone deprived media for 5 days, before growing another 24h in the absence (−E2) or presence of 10 nM E2 and/or 1 μM fulvestrant as indicated. One step quantitative RT-PCR was performed using iScript reverse transcriptase (Bio-Rad) with 25 ng RNA. Expression was normalized to GAPDH and relative expression was calculated as fold change using the 2^−ΔΔCt^ method with YFP −E2 set to 1.

#### Immunohistochemistry

IHC staining was performed with assistance from The Lester and Sue Smith Breast Center Pathology Core at Baylor College of Medicine (Houston, TX, USA). Tissue sections were incubated at 58°C overnight in a dry slide incubator and deparaffinized in xylene and graded alcohol washes. Antigen retrieval was performed in 0.1 M Tris-HCl pH 9.0 following by quenching in 3% H_2_O_2_. The following antibodies were used to stain for 1h at RT: ERa (clone 6F11, Novocastra, 1:200), pRb (Ser780) (clone D59B7, Cell Signaling, 1:25), Ki67 (clone MIB-1, Dako, 1:200), and PR (clone PgR 1294, Dako, 1:1600). After washing in TBS, EnVision labeled polymer-HRP antimouse or anti-rabbit antibodies (Dako) were added for 30 min. at RT. Slides were washed with TBS then developed with DAB+ solution (Dako) and DAB sparkle enhancer (Biocare). After washing in TBS, slides were counstained with Hematoxylin, dehydrated, and cleared before coverslipping with Cytoseal (VWR). ER positive staining cells were quantified in lung sections from 5 T47D xenograft bearing mice. Stained WHIM18 tumor and lung sections were quantified from 5 mice per treatment group.

#### Scratch Wound Assay

Stable T47D cells were hormone deprived for 7 days before seeding in hormone deprived media at 50,000 cell/well in a 96-well ImageLock plate (Essen BioScience). The following day, cells were treated 10 μg/mL mitomycin C (Sigma) for 2h before wounding with a WoundMaker (Essen BioScience). Cells were washed with hormone deprived media then fresh hormone deprived media containing mitomycin C was added. Images were acquired every 3h for 72h with an IncuCyte live-cell analysis system (Essen BioScience). Fresh hormone deprived media plus mitomycin C was changed every 24h. Cell motility assessed by the relative wound density (RWD) calculated by measuring density in the wound area relative to the density outside the wound area at 72h. The RWD is 0% at 0h and 100% when the density inside the wound is the same as the density outside the wound, therefore normalizing for changes in density due to proliferation outside the wound. Representative images are depicted and quantification from average of three independent experiments ± SEM are shown. *P*-values based on ANOVA followed by Dunnett’s post hoc test for multiple comparisons correction.

#### Immunofluorescence Microscopy

Hormone deprived stable T47D cells were seeded onto poly-D-lysine coated coverslips (Fisher) and grown overnight. Cells were fixed with 4% formaldehyde for 20 min. at RT followed by permeabilization with 0.2% Triton X-100 for 10 min at RT and blocking with 10% normal goat serum for 1h. Antibodies against E-cadherin (cat# 14472, Cell Signaling, 1:50), vimentin (cat# 5741, Cell Signaling, 1:100) or HA-tag (cat# 2367, Cell Signaling, 1:50) were incubated overnight at 4°C then goat anti-mouse-488 (cat# A-11011, Invitrogen, 1:1000), goat anti-rabbit-488 (cat# A-11008, Invitrogen, 1:1000), or goat anti-mouse-568 (cat# A-11004, Invitrogen, 1:1000) was added for 30 min at RT. Coverslips were mounted onto slides with ProLong Gold Antifade Reagent (Invitrogen). Fluorescence images were acquired on a Nikon Eclipse Ti microscope equipped with a CoolSNAP EZ camera (Photometrics Scientific) using a Plan Apo 40X/0.95 aperture objective and Nikon NIS elements software. Images were quantified with ImageJ by setting a threshold from E-cadherin fluorescence channel from ESR1-WT cells which gave cell surface appearance. The same threshold was applied to images acquired from all other cell lines and cells were considered E-cadherin^+^ when cell surface signal was present using the described threshold. 2-3 images per cell line were quantified and shown are averages from two independent experiments ± SEM.

## QUANTIFICATION AND STATISTICAL ANALYSIS

All statistical tests were performed with GraphPad Prism 7. *P*-values less than 0.05 were considered statistically significant (*p<0.05, ** p<0.01, *** p<0.001, **** p<0.0001). For box and whiskers plots, the box depicts interquartile range with median line and whiskers extending to minimum and maximum values for each group.

Immunofluorescence images were quantified with ImageJ by setting a threshold from E-cadherin fluorescence channel from ESR1-WT cells which gave cell surface appearance. The same threshold was applied to images acquired from all other cell lines and cells were considered E-cadherin^+^ when cell surface signal was present using the described threshold. 2-3 images per cell line were quantified and shown are averages from two independent experiments ± SEM.

For cell proliferation assays, significance was determined based on one-way ANOVA followed by Tukey’s post hoc test for multiple comparisons correction for ESR1-e6>YAP1 or ESR1-e6>PCDH11X fusion-expressing cells compared to all other stable T47D cells within a treatment group (indicated by asterisks) or using two-way ANOVA followed by Bonferonni’s post hoc test for multiple comparisons correction for each construct after E2 stimulation, +E2 versus −E2 (#### p < 0.0001). Data are mean ± SEM of three independent experiments. For palbociclib sensitivity assays in stable T47D and MCF7 cell lines, each point represents averages ± SEM from 3-4 independent experiments of relative cell growth for indicated palbociclib dose, calculated by taking the palbociclib treated Day 12/Day 1 alamarBlue reading ratio divided by vehicle treated Day 12/Day 1 ratio. *P*-values describes significance between YFP +E2, ESR1-e6>YAP1, and ESR1-e6>PCDH11x slopes compared to YFP −E2 as measured by ANOVA with Tukey’s post hoc analysis for multiple comparisons.

For ChIP-qPCR assays, bar graphs depict enrichment of ER binding regions in hormone deprived stable T47D cells before and after stimulation with E2 (100 nM) for 45 min as determined by HA-ChIP followed by qPCR for ER binding regions of estrogen responsive genes as indicated and negative ER binding region. Average values from 3 experiments are shown ± SEM. Asterisks denote significant differences in binding compared to WT-ER −E2 for each gene binding region as determined by ANOVA followed by Tukey’s post hoc test.

### *In Vivo* Analysis

For T47D xenograft assays, significance of tumor volumes Day 146 post injection was determined based on Kruskal-Wallis test followed by Dunn’s post hoc analysis for multiple comparisons correction comparing YFP −E2 to all other groups with N = 6 mice per group. For ER^+^ cell counting in the lungs, ER^+^ cells from IHC images of 5 mice bearing xenografted tumors at Day 146 were manually counted. Statistical analysis was based on Kruskal-Wallis test with Dunn’s post hoc analysis for multiple comparisons correction comparing YFP versus fusion-bearing groups and YFP +E2.

For WHIM18 PDX assays, Figure 5B depicts averages of tumor volumes from 8-11 mice per group ± SEM are shown. *P*-value determined by unpaired t test describes significance of tumor growth rates (slopes) derived from tumor volumes at day of randomization/start of treatment (Day 61 post transplantation) to experiment end (Day 91 post transplantation) for vehicle and palbociclib treated mice. Figure S5C depicts tumor growth rates as described above for all tumors measured in each condition. Middle line represents mean tumor volume ± SD. Day 0 post treatment is the same as treatment start/Day 61 post transplantation and represents the tumor growth rate from time tumors were palpable (Day 49 post transplantation) up to treatment start date. Day 30 is the same as Day 91 post transplantation and represents on-treatment tumor growth rates. *P*-values determined by one-way ANOVA with Tukey’s post hoc analysis for multiple comparisons. For IHC images, positive staining cells were quantified in tumor and lung sections from 5 mice per treatment group. Bar graphs represents mean ± SD and *P*-values indicate significance as determined by Wilcoxon rank-sum tests.

### ChIP-seq Analysis

Single-end 50 bp reads were aligned to hg19 (GRCh37) reference genome using BWA(Li and Durbin, 2010) and alignment files were converted to BED format using BEDTools (Quinlan and Hall, 2010). BED files were used for peak calling by MACS v1.4.2 (Zhang et al., 2008). MACS peaks (p < 1e^−7^ cutoff and associated FDRs) were annotated with GREAT (McLean et al., 2010) using default settings. Motif analysis was performed by taking ~100 bp sequences centered on the summit of peaks and submitted for enrichment analysis using MEME-ChIP in normal mode (Bailey and Elkan, 1994). *P*-values represents the probability that an equal or better site would be found in a random sequence of the same length conforming to the background letter frequencies (Bailey and Elkan, 1994).

### RNA-seq Analysis

Paired-end 100 bp reads were aligned to hg19 (GRCh37) reference genome using RSEM v1.2.31 (Li and Dewey, 2011) and Bowtie 2 (Langmead and Salzberg, 2012). TPM (Transcripts Per Million) values calculated by RSEM were log2 transformed and row Z-scores were generated for the all heatmaps shown. Differential gene expression analysis was performed using EBseq (Leng et al., 2013) with FDR < 0.1 as a cutoff comparing 4 groups: (1) YFP +E2 versus YFP −E2, (2) ESR1-e6>YAP1 −E2 versus YFP −E2, (3) ESR1-e6>PCDH11X −E2 versus YFP −E2, and (4) ESR1-e6>NOP2 −E2 versus YFP −E2. Hierarchal clustering was performed on differentially expressed genes for which a nearby binding site within 1 Mb was observed by ChIP-seq shared by ESR1-WT, ESR1-e6>YAP1, ESR1-e6>PCDH11X, and ESR1-e6>NOP2 for Figure 3C. Clustering was also performed on differentially expressed genes for which a nearby site within 1 Mb was selectively bound by both ESR1-e6>YAP1 and ESR1-e6>PCDH11X but bound by ESR1-WT nor ESR1-e6>NOP2 (Figure S4C).

## DATA SOFTWARE AND AVAILABILITY

The accession number for the ChIP and RNA sequencing data from T47D reported in this paper is GEO: GSE116170. TCGA data for fusion gene discovery and for gene expression analysis can be downloaded from https://portal.gdc.cancer.gov/ and https://portal.gdc.cancer.gov/legacy-archive. RNA-seq of human primary breast tumors from two neoadjuvant aromatase inhibitor clinical trials can accessed through dbGaP phs000472.

## Supplementary Material

Suppl. Figures

Table S2

Table S3

## Figures and Tables

**Table:** KEY RESOURCES TABLE

REAGENT or RESOURCE	SOURCE	IDENTIFIER

Antibodies		
Rabbit monoclonal anti-HA (clone C29F4)	Cell Signaling Technology	Cat#3724; RRID:AB_1549585
Mouse monoclonal anti-HA (clone 6E2)	Cell Signaling Technology	Cat#2367; RRID:AB_331789
Mouse monoclonal anti-HA (clone F-7)	Santa Cruz Biotechnology	Cat#sc-7392; RRID:AB_627809
Rabbit monoclonal anti-ERα (clone 60C), N-terminal	Millipore	Cat#04-820; RRID:AB_1587018
Rabbit polyclonal anti-ERα, C-terminal	Santa Cruz Biotechnology	Cat#sc-543; RRID:AB_631471
Mouse monoclonal anti-ERα (clone 6F11)	Leica Microsystems	Cat#NCL-L-ER-6F11; RRID:AB_563706
Mouse monoclonal anti-β-Actin	Sigma-Aldrich	Cat#A5316; RRID:AB_476743
Mouse monoclonal anti-E-Cadherin (clone 4A2)	Cell Signaling Technology	Cat#14472; RRID:AB_2728770
Rabbit monoclonal anti-Vimentin (clone D21H3)	Cell Signaling Technology	Cat#5741; RRID:AB_10695459
Rabbit monoclonal anti-Snail (clone C15D3)	Cell Signaling Technology	Cat#3879; RRID:AB_2255011
Rabbit monoclonal anti-Phospho-Rb (Ser780) (clone D59B7)	Cell Signaling Technology	Cat#8180; RRID:AB_10950972
Mouse monoclonal anti-Ki-67 (MIB-1) (clone Ki-67)	Beckman Coulter	Cat#IM1316; RRID:AB_131615
Mouse monoclonal anti-PR (clone PgR 1294)	Dako	Cat#M3568; RRID:AB_2252608

Biological Samples		
WHIM18 patient-derived xenograft (PDX)	Li et al., 2013	N/A

Chemicals, Peptides, and Recombinant Proteins		
β-Estradiol (E2)	Sigma-Aldrich	Cat#E4389
Fulvestrant	Selleckchem	Cat#S1191
Pablociclib	Pfzier and Selleckchem	N/A and Cat#1116

Critical Commercial Assays		
Dual-Luciferase Reporter Assay System	Promega	Cat#1910

Deposited Data		
Human reference genome NCBI build 37, GRCh37	Genome Reference Consortium	http://www.ncbi.nlm.nih.gov/projects/genome/assembly/grc/human
WGS and RNA-seq of WHIM18 PDX	Li et al., 2013	dbGaP: phs000611
RNA-seq of human primary breast tumors from TCGA	Ciriello et al., 2015	https://portal.gdc.cancer.gov/ and https://portal.gdc.cancer.gov/legacy-archive
RNA-seq of human primary breast tumors from two neoadjuvant aromatase inhibitor clinical trials (Z1031/POL)	Olson et al., 2009 and Ellis et al., 2011	dbGaP: phs000472
ChIP-seq and RNA-seq from T47D cell lines	This paper	GEO: GSE116170

Experimental Models: Cell Lines		
Human: HEK293T	ATCC	CRL-3216
Human: T47D	ATCC	HTB-133
Human: MCF7	ATCC	HTB-22

Experimental Models: Organisms/Strains		
*NOD-SCID*-IL2R_γc_^−/−^ mice	Jackson Laboratories	Cat#005557
Fox Chase SCID Beige mice	Charles River	N/A

Oligonucleotides		
See Table S4 for sequences of mRNA-qPCR primers, ChIP-qPCR primers, and siRNA	N/A	N/A

Recombinant DNA		
pFLRu-FH	Li et al., 2013	N/A
pCDH-CMV-MCS-EF1α-RFP+Puro	System Biosciences	Cat#CD516B-2
pCDH-CMV-MCS-EF1α-Puro	System Biosciences	Cat#CD510B-1
3X ERE TATA luc	Hall and McDonnell, 1999	Addgene Plasmid Cat#11354
pGL4.70[*hRluc*]	Promega	Cat#E6881; GenBank AY738226

Software and Algorithms		
BWA	Li and Durbin, 2010	N/A
BEDTools	Quinlan and Hall, 2010	N/A
GREAT	McLean et al., 2010	N/A
MEME-ChIP	Bailey and Elkan, 1994	N/A
RSEM 1.2.31	Li and Dewey, 2011	N/A
Bowtie 2	Langmead and Salzberg, 2012	N/A
EBseq	Leng et al., 2013	N/A
GraphPad Prism 7	GraphPad Software	N/A
ImageJ	NIH	https://imagej.nih.gov/ij/

## Highlights

- *ESR1* fusions drive ligand-independent growth and endocrine therapy resistance
- *ESR1* fusions reprogram the ER cistrome to drive EMT and metastasis
- CDK4/6 inhibition suppresses *ESR1* fusion-induced growth
