# Supplementary material for: Functional Annotation of ESR1 Gene Fusions in Estrogen Receptor-Positive Breast Cancer
Source: Cell Rep. Author manuscript; Available in PMC 2018 Oct 4. (PMC6171747; doi:10.1016/j.celrep.2018.07.009)
Supplement: Suppl. Figures [file NIHMS985142-supplement-Suppl__Figures.pdf]

Cell Reports, Volume *24*

Supplemental Information

Functional Annotation of *ESR1* Gene Fusions

in Estrogen Receptor-Positive Breast Cancer

Jonathan T. Lei, Jieya Shao, Jin Zhang, Michael Iglesia, Doug W. Chan, Jin Cao, Meenakshi Anurag, Purba Singh, Xiaping He, Yoshimasa Kosaka, Ryoichi Matsunuma, Robert Crowder, Jeremy Hoog, Chanpheng Phommaly, Rodrigo Goncalves, Susana Ramalho, Raquel Mary Rodrigues Peres, Nindo Punturi, Cheryl Schmidt, Alex Bartram, Eric Jou, Vaishnavi Devarakonda, Kimberly R. Holloway, W. Victoria Lai, Oliver Hampton, Anna Rogers, Ethan Tobias, Poojan A. Parikh, Sherri R. Davies, Shunqiang Li, Cynthia X. Ma, Vera J. Suman, Kelly K. Hunt, Mark A. Watson, Katherine A. Hoadley, E. Aubrey Thompson, Xi Chen, Shyam M. Kavuri, Chad J. Creighton, Christopher A. Maher, Charles M. Perou, Svasti Haricharan, and Matthew J. Ellis

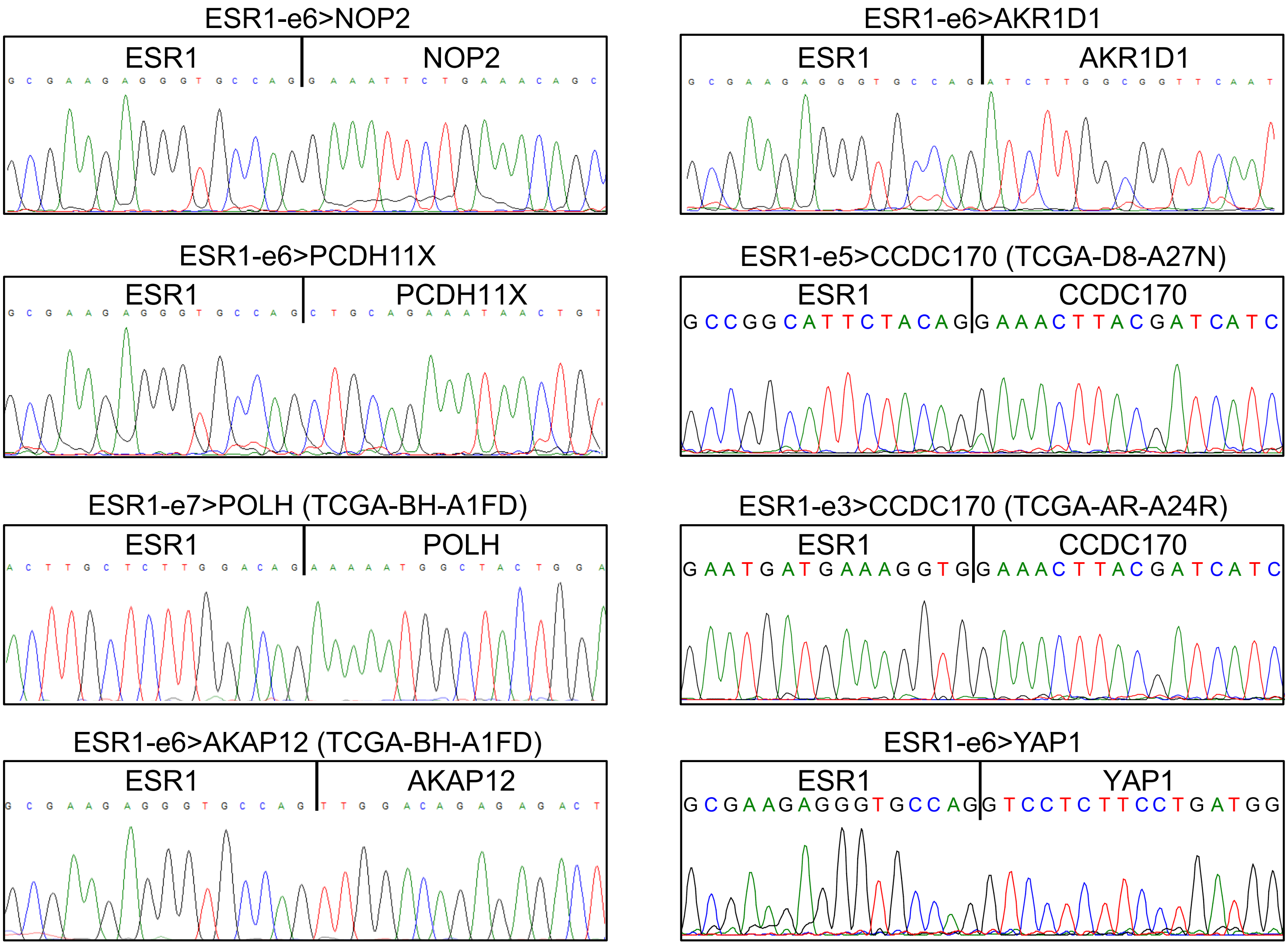

Figure S1 related to Figure 1. PCR validation of *ESR1* fusions. cDNAs were synthesized from patient RNA or WHIM18 tumor RNA, followed by PCR amplification and sanger sequencing. Black line indicates fusion break point. Sequences contributed by *ESR1* are to the left of the breakpoint and from fusion partner sequences to the right of the breakpoint.

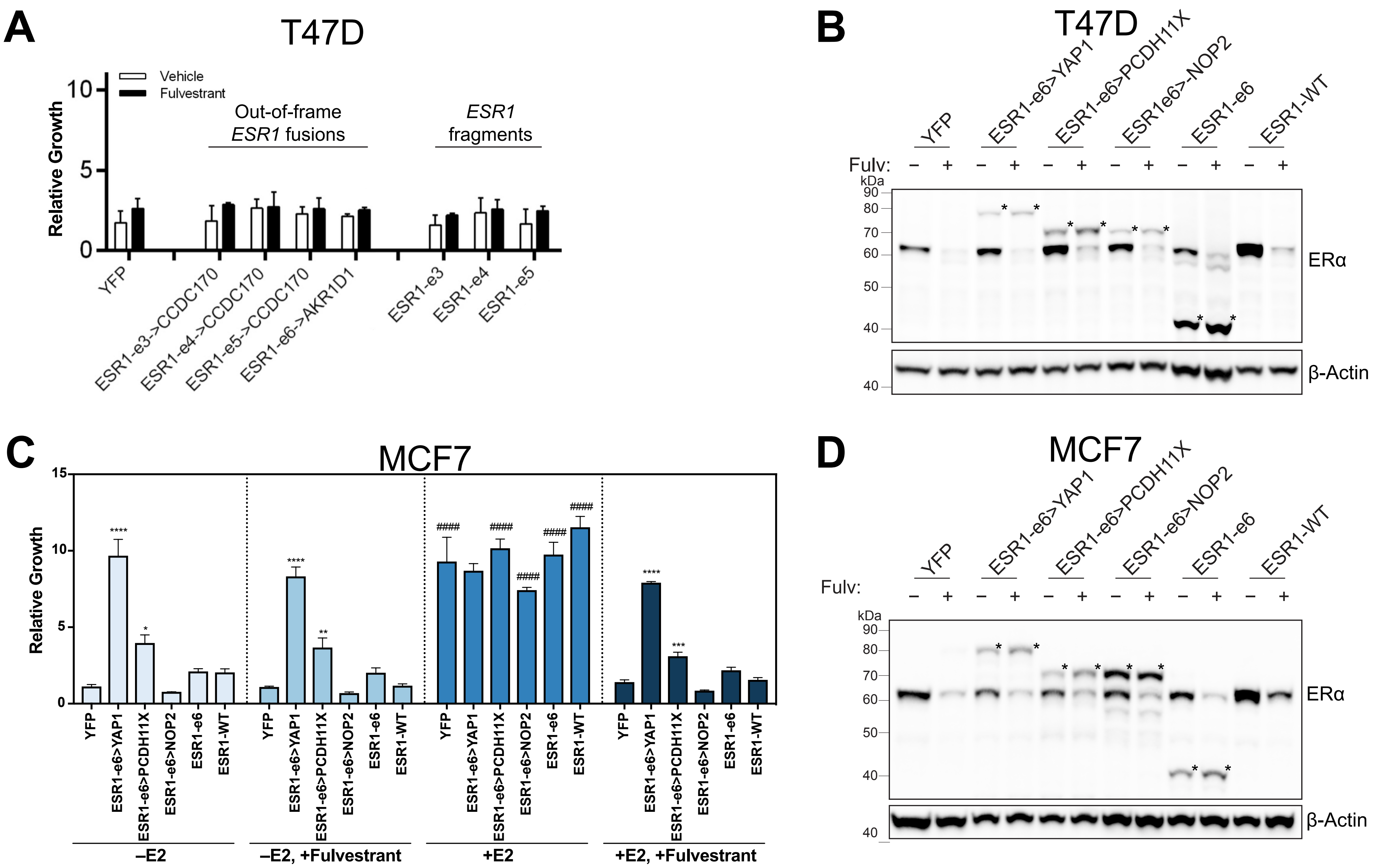

Figure S2 related to Figure 2. Out-of-frame *ESR1* fusions lack estrogen-independent growth promoting ability and in-frame *ESR1* fusions from endocrine refractory disease promotes estrogen-independent and fulvestrant-resistant growth. (**A**) Bar graphs depicting growth of hormone deprived stable out-of-frame *ESR1* fusion or *ESR1* fragments expressing T47D cells in the absence (open bars) or presence of fulvestrant (black bars). Data are average of 3 independent experiments ±SEM. (**B**) Western blotting of ERα in hormone deprived stable T47D cells treated in the absence of presence of fulvestrant. Asterisks denote *ESR1* fusion or ESR1-e6. β-Actin used as loading control. (**C**) Similar to Figure 2C, except hormone deprived stable MCF7 cells expressing the indicated constructs were used. Significance was determined based on ANOVA followed by Tukey's post-hoc test for multiple comparisons correction for ESR1-e6>YAP1 or ESR1-e6>PCDH11X fusion expressing cells compared to all other stable T47D cells within a treatment group (**** $p < 0.0001$, ** $p < 0.01$, * $p < 0.05$) or using two-way ANOVA followed by Bonferonni's post-hoc test for multiple comparisons correction for each construct after E2 stimulation, –E2 vs +E2 (#### $p < 0.0001$). Data are mean ± SEM of three independent experiments. (**D**) Similar to (**B**) except hormone deprived stable MCF7 cells were used.

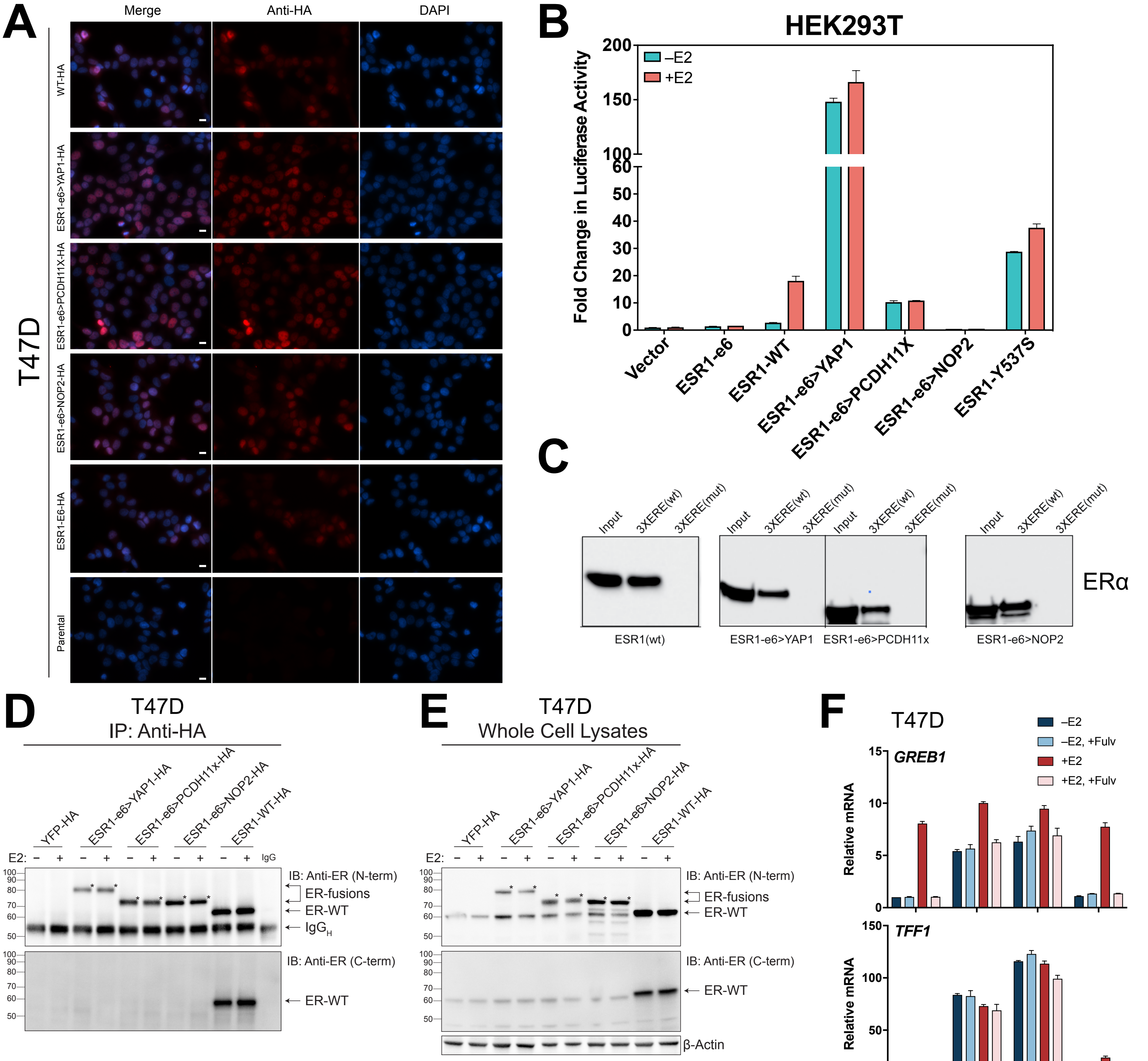

Figure S3 related to Figure 3. In-frame *ESR1* fusions bind EREs but have differential abilities to drive transcription. (**A**) Immunofluorescence staining with Anti-HA antibody, pseudocolored in red, was performed on hormone-deprived stable T47D cells as indicated, showing nuclear localization of all HA-tagged ESR1-WT, *ESR1* fusions, and ESR1-e6 truncation. Parental T47D cells lacking exogenous expression of any construct was used as a negative control. DAPI stained nuclei pseudocolored in blue. 10 µm scale bar. (**B**) Bar graphs depicting ERE-luciferase reporter activity in hormone-deprived HEK-293T cells ±2.5 nM E2 for 24h. Averages of Firefly/Renilla luminescence readings from each sample were calculated and expressed as fold change in activity relative to Vector transfected –E2. Shown are the averages of two independent experiments ±SEM. (**C**) Full-length ESR1(wt) or *ESR1* fusions were transfected into HEK293T cells and subjected to pulldown with 3X ERE(wt) or ERE(mut) containing a DNA binding inactivating double zinc finger mutations as negative controls and analyzed by western blotting using a N-terminal ER antibody along with 10% input as positive controls. (**D**) Lysates from hormone deprived stable T47D cells stimulated with E2 (15 min) were immunoprecipitated with an HA antibody or rabbit IgG control then blotted with N-terminal ERα antibody demonstrating successful IP of fusion ER (asterisks) and WT-ER (top panel). Blotting with a C-terminal ERα antibody that recognizes only WT-ER detects strong co-IP with WT-ER but lack of WT-ER co-IP with ER fusions. (**E**) Whole cell lysates (inputs) analyzed by Western blot from (**C**) with N- and C-terminal ER antibodies along with β-Actin control. (**F**) Same as Figure 3D, except –E2 and +E2 conditions shown for all cell lines analyzed.

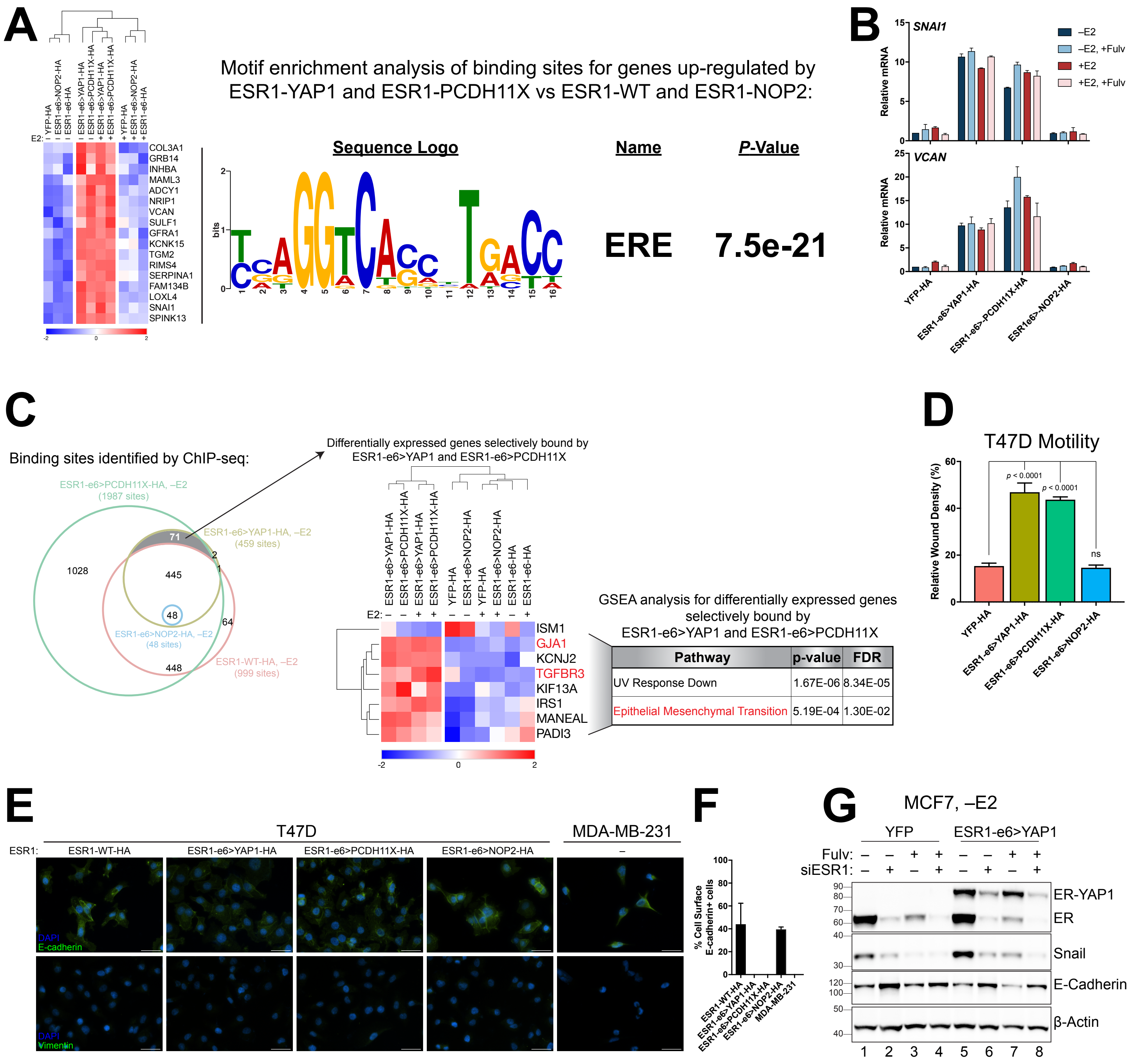

Figure S4 related to Figure 4. Active *ESR1* fusions promote metastasis by up-regulating an EMT-like transcriptional program. (**A**) MEME-ChIP motif enrichment was performed using 100 bp sequences centered around each peak's summit (derived from Table S3) for genes strongly upregulated by the active ESR1-e6>YAP1 and ESR1-e6>PCDH11X fusions (Figure 4A) demonstrating ERE motif enrichment with overall *P*-value of 7.5e-21. (**B**) Same as in Figure 4C, except ±E2 conditions shown for all T47D cell lines analyzed. Data are averages of two independent experiments ±SEM. (**C**) Hierarchal clustering was performed on differentially expressed genes within 1Mb of 71 selectively bound sites by ESR1-e6>YAP1 and ESR1-e6>PCDH11X (shaded gray overlap). GSEA hallmark pathway analysis for these genes demonstrates enrichment for UV response and EMT hallmark pathways. *GJA1* and *TGFBR3* (shown in red) contributed to EMT pathway enrichment. Scale bar indicates row Z-score. (**D**) Quantification of relative wound density 72h post wounding from Figure 4E. Data are averages from three independent experiments ±SEM. (**E**) Representative immunofluorescence images of two independent experiments from hormone deprived stable T47D cells expressing constructs as indicated and hormone deprived MDA-MB-231 stained with E-cadherin and vimentin antibodies pseudocolored in green and DAPI stained nuclei pseudocolored in blue. 50 µm scale bar. (**F**) Bar graphs depicting average number of cell surface E-cadherin positive (E-cadherin+) cells quantified from images in (**E**). Data are averages of two independent experiments ±SEM. (**G**) Same as in Figure 4F, except using stable MCF7 cell lines.

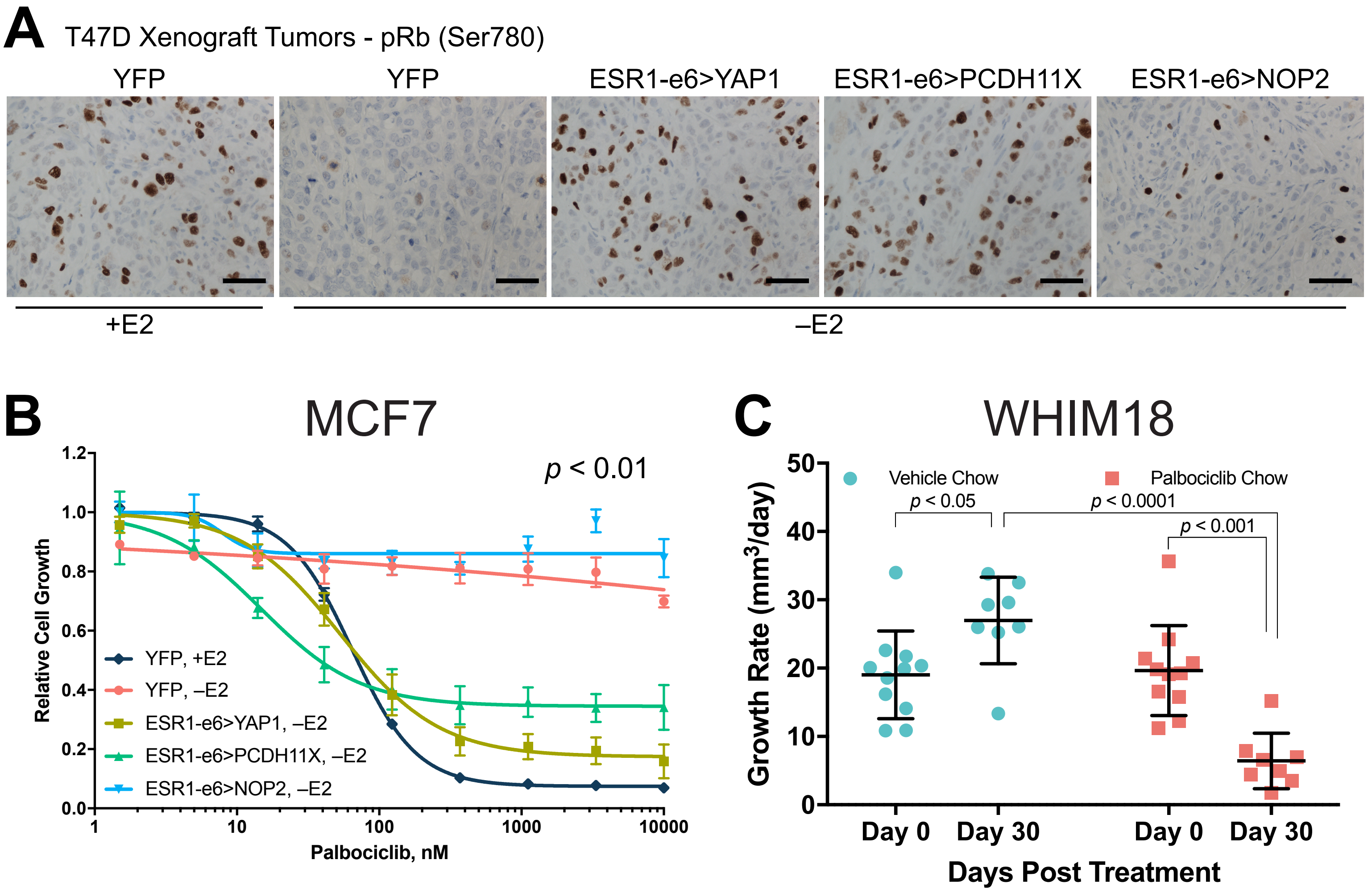

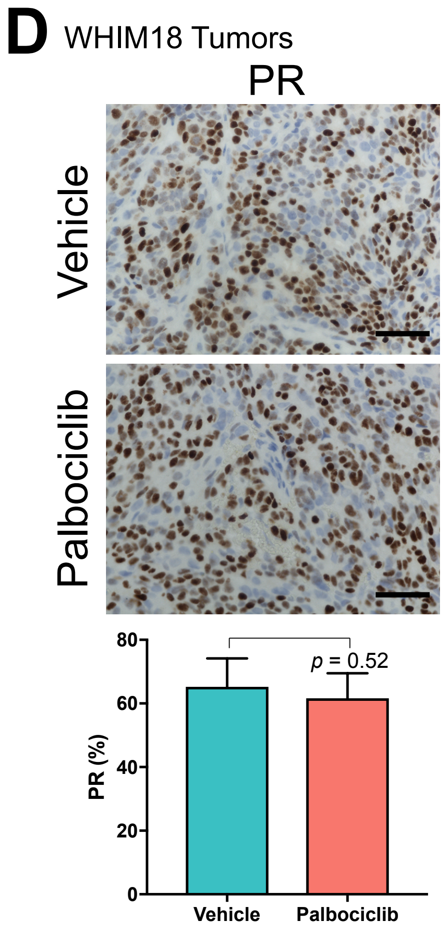

Figure S5 related to Figure 5. ***ESR1*** **fusions induce cell cycle activity through activation of Rb and** ***ESR1*** **fusion driven growth can be suppressed with CDK4/6 inhibitor treatment.** (**A**) IHC images of T47D xenograft tumor sections with indicated constructs grown in the absence or presence of E2 supplementation stained with a phospho-Rb (pRb) (Ser780) antibody. 100 µm scale bar. (**B**) Relative growth of hormone deprived stable MCF7 cells treated with increasing concentrations of a CDK4/6 inhibitor, palbociclib, and in the presence of E2 for YFP (+E2) normalized to vehicle treated cells for each condition. *P*-value describes significance between YFP +E2, ESR1-e6>YAP1, and ESR1-e6>PCDH11X slopes compared to YFP –E2 as measured by ANOVA with Tukey's post-hoc analysis for multiple comparisons. Data shown are averages of three independent experiments ±SEM. (**C**) Day 0 post treatment WHIM18 tumor growth rates were calculated from slopes of tumor growth from Day 49 to Day 61 post tumor transplantation. Day 30 post treatment growth rate was calculated from Day 61 to Day 91 post tumor transplantation. Individual tumor growth rates from each mouse are plotted with middle line representing median value and extending from 25% to 75% percentile values. *P*-values determined by two way ANOVA with Tukey's post-hoc analysis for multiple comparisons correction. (**D**) Progesterone receptor (PR) expression was examined by IHC in WHIM18 tumor sections from vehicle or palbociclib treated mice demonstrating that palbociclib does not alter PR levels. Quantification of IHC staining below with Wilcoxon rank-sum test used to calculate significance comparing treatment groups. Data are averages counts from 5 tumor sections from each treatment group with error bars representing SD. 100 µm scale bar.

N-term ESR1 aa (5' exons)	Study (Sample)	Fusion Transcript	Source	Frame	Intra- OR Inter-Chr	Stage	PAM50	TP53	BRCA2	PIK3CA	AKT1	PTEN	KRAS	MLL3	GATA3	MAP3K1	CDH1	Platform
(e2)	TCGA-E9-A1NA	ESR1->C6orf211	1° tumor	Out of-frame	INTRA	IIA	LumB	WT	WT	WT	WT	WT	WT	WT	D335Gfs*	WT	WT	WES
	TCGA-A2-A0YG	ESR1->CCDC170	1° tumor	Out-of-frame	INTRA	IIIC	LumB	X125 splice	WT	AMP	WT	DEL	WT	WT	WT	WT	WT	WES
	TCGA-A2-A0CT	ESR1->CCDC170	1° tumor	Out-of-frame	INTRA	IIA	LumB	WT	WT	WT	WT	WT	WT	WT	WT	WT	WT	WES
151aa (e3)	TCGA-AR-A24R	ESR1->CCDC170	1° tumor	Out-of-frame	INTRA	IIIA	LumB	WT	WT	WT	WT	WT	WT	WT	WT	WT	WT	WES
	TCGA-AQ-A04L	ESR1->UTRN	1° tumor	Out-of-frame	INTRA	IIA	LumA	H193R	WT	WT	WT	WT	WT	WT	WT	WT	WT	WES
	NeoAI Trials (17387)	ESR1->CCDC170	1° tumor	Out-of-frame	INTRA	IIA	LumB	WT	L2745fs	N/A	WT	WT	WT	WT	WT	WT	WT	WES
214aa (e4)	TCGA-BH-A1FD	ESR1->CCDC170	1° tumor	Out-of-frame	INTRA	I	LumB	WT	WT	WT	WT	WT	WT	WT	WT	WT	WT	WES
215aa (e4)	TCGA-BH-A1EV	ESR1->BNC2	1° tumor	Out-of-frame	INTER	IIIA	Her2	WT	AMP	WT	WT	WT	AMP	WT	WT	WT	WT	WES
	TCGA-A8-A06Q	ESR1->USP25	1° tumor	Out-of-frame	INTER	IIIA	LumB	C238Y	WT	H1047R	WT	WT	WT	WT	WT	WT	WT	WES
253aa (e5)	TCGA-D8-A27N	ESR1->CCDC170	1° tumor	Out-of-frame	INTRA	IIIA	LumB	R175H	WT	AMP	WT	WT	WT	WT	WT	WT	WT	WES
	NeoAI Trials (16002)	ESR1->GPR126	1° tumor	Out-of-frame	INTRA	IIA	LumB	WT	WT	D454N	WT	DEL	WT	WT	WT	WT	WT	WES
	NeoAI Trials (814)	ESR1->PCMT1	1° tumor	Out-of-frame	INTRA	IIB	LumB	WT	WT	WT	WT	WT	WT	R4139	WT	WT	WT	WES
	NeoAI Trials (16315)	ESR1->CCDC170	1° tumor	Out-of-frame	INTRA	IIIB	LumB	WT	WT	GNR106 del	WT	WT	WT	WT	WT	WT	WT	WES
365aa (e6)	NeoAI Trials (16986)	ESR1->AKR1D1	1° tumor	Out-of-frame	INTER	N/A	LumB	WT	WT	WT	WT	WT	WT	T316S	WT	WT	WT	WES
	NeoAI Trials (17502)	ESR1->NOP2	1° tumor	In-frame	INTER	N/A	LumB	H179R	WT	WT	WT	WT	WT	WT	WT	WT	WT	WES
	Late Stage (WHIM18)	ESR1->YAP1	PDX from metastatic patient	In-frame	INTER	IV	LumB	WT	WT	E545K	WT	WT	WT	WT	WT	WT	WT	WGS
	Late Stage (360)	ESR1->PCDH11X	Chest wall recurrence	In-frame	INTER	IV	LumB	N/A	N/A	N/A	N/A	N/A	N/A	N/A	N/A	N/A	N/A	N/A
	TCGA-BH-A1FD	ESR1->AKAP12	1° tumor	In-frame	INTRA	I	LumB	WT	WT	WT	WT	WT	WT	WT	WT	WT	WT	WES
412aa (e7)	TCGA-BH-A1FD	ESR1->POLH	1° tumor	In-frame	INTRA	I	LumB	WT	WT	WT	WT	WT	WT	WT	WT	WT	WT	WES

C-term ESR1 aa (3' exons)	Study (Sample)	Fusion Transcript	Source	Frame	Intra- OR Inter-Chr	Stage	PAM50	TP53	BRCA2	PIK3CA	AKT1	PTEN	KRAS	MLL3	GATA3	MAP3K1	CDH1	Platform
(e9)	TCGA-A8-A08I	AKAP7->ESR1	1° tumor	Out-of-frame	INTRA	IIA	LumB	Y163C	DEL	WT	DEL	WT	WT	WT	WT	WT	WT	WES
(e10)	TCGA-A8-A08I	AKAP7->ESR1	1° tumor	Out-of-frame	INTRA	IIA	LumB	Y163C	DEL	WT	DEL	WT	WT	WT	WT	WT	WT	WES

Table S1. Related to Figure 1 and Table S2. Summary of *ESR1* fusion transcripts from ER+ samples. The TCGA dataset includes 728 breast cancer patients (Ciriello et al., 2015). The NeoAI Trials dataset includes 41 aromatase inhibitor sensitive neoadjuvant primary samples, 40 aromatase inhibitor resistant neoadjuvant primary samples (Ellis et al., 2011; Olson et al., 2009). The Late Stage dataset includes 25 advanced ER+ endocrine therapy refractory, metastatic biopsy samples (Table S2) and includes WHIM18, a PDX derived from a metastatic biopsy from a patient with endocrine therapy resistant disease (Li et al., 2013). ChimeraScan (Iyer et al., 2011) and INTEGRATE (Zhang et al., 2016) were used to detect gene fusions in RNA-seq data and in some cases with whole genome data. *ESR1* fusions are presented according to the number of 5' exons (top portion of table) or 3' exons (bottom portion of table) retained in each of the indicated *ESR1* fusions with corresponding amino acids (aa). The first two 5' exons of *ESR1* (e2) are non-coding exons. The -> indicates direction of fusion transcript from 5' to 3' direction. Also shown are mutational status of genes found to be significantly mutated in ER+ breast cancer representing common risk factors for hormone receptor positive breast cancer (Ellis et al., 2012) and platform used to determine mutational status (WES, whole-exome sequencing; WGS, whole-genome sequencing).

Oligonucleotides		
mRNA-qPCR primers:	Reference:	Identifier:
GREB1: Forward, 5'-CAAAGAATAACCTGTTGGCCCTGC-3'	This paper	N/A
GREB1: Reverse, 5'-GACATGCCTGCGCTCTCATACTTA-3'		
TFF1: Forward, 5'-GTGTCACGCCCTCCCAGT-3'	This paper	N/A
TFF1: Reverse, 5'-GGACCCCACGAACGGTG-3'		
PGR: Forward, 5'-CTTAATCAACTAGGCGAGAG-3'	This paper	N/A
PGR: Reverse, 5'-AAGCTCATCCAAGAATACTG-3'		
SNAI1: Forward, 5'-TCGGAAGCCTAACTACAGCGA-3'	This paper	N/A
SNAI1: Reverse, 5'-AGATGAGCATTGGCAGCGAG-3'		
VCAN: Forward, 5'-CCAGTGTGAACTTGATTTTG-3'	Sigma-Aldrich	FH1_VCAN
VCAN: Reverse, 5'-CAACATAACTTGGAAGGCAG-3'	Sigma-Aldrich	RH1_VCAN
GAPDH: Forward, 5'-CTTTTGCGTCGCCAG-3'	Sigma-Aldrich	FH2_GAPDH
GAPDH: Reverse, 5'-TTGATGGCAACAATATCCAC-3'	Sigma-Aldrich	RH2_GAPDH
ChIP-qPCR primers:		
GREB1 ERE: Forward, 5'-AGCAGTGAAAAAAAGTGTGGCAACTGGG-3'	Lin et al., 2004	N/A
GREB1 ERE: Reverse, 5'-CGACCCACAGAAATGAAAAGGCAGCAAACT-3'		
TFF1 ERE3: Forward, 5'-GTCGTTGCCAGCGTTTCC-3'	This paper	N/A
TFF1 ERE3: Reverse, 3'-CTTCTCCACGCCCTGTAAATTT-3'		
PGR Enhancer: Forward, 5'-GATGACAGAAGGAGAAGTTAGAAG-3'	This paper	N/A
PGR Enhancer: Reverse, 5'-ATATGGCATTGAAGCAACAGG-3'		
Chr20 negative region: Forward, 5'-GAGGCTGTGCTTGGAGTAGG-3'	Carroll et al., 2006	N/A
Chr20 negative region: Reverse, 3'-CGTTTCCCCTGTGAAAGGTA-3'		
siESR1: Sense, 5'-GAAAGAUUGGCCAGUACCA-3'	Sigma-Aldrich	Oligo#3020649250-000030
siESR1: Antisense, 5'-UGGUACUGGCCAAUCUUUC-3'	Sigma-Aldrich	Oligo#3020649250-000040

Table S4. Sequences of mRNA-qPCR and ChIP-qPCR primers, and siRNA. Related to STAR Methods.
